# Author Correction: Acceptor engineering for NIR-II dyes with high photochemical and biomedical performance

**DOI:** 10.1038/s41467-022-32802-2

**Published:** 2022-08-25

**Authors:** Aiyan Ji, Hongyue Lou, Chunrong Qu, Wanglong Lu, Yifan Hao, Jiafeng Li, Yuyang Wu, Tonghang Chang, Hao Chen, Zhen Cheng

**Affiliations:** 1 State Key Laboratory of Drug Research, Molecular Imaging Center, Shanghai Institute of Materia Medica, Chinese Academy of Sciences, Shanghai, 201203 China; 2 Shanghai Institute of Technical Physics of the Chinese Academy of Sciences, Shanghai, 200083 China; 3 University of Chinese Academy of Sciences, No.19A Yuquan Road, Beijing, 100049 China; 4 Bohai rim Advanced Research Institute for Drug Discovery, Yantai, Shandong, 264117 China

**Keywords:** Fluorescent dyes, Imaging studies, Fluorescence imaging

Correction to: *Nature Communications* 10.1038/s41467-022-31521-y, published online 02 July 2022

The original version of this Article contained an error in the figure legend of Figure 2L where “TPA-TQT” was mistakenly written as “TPA-BBT”. This has been corrected in both the PDF and HTML versions of the Article.

