# Supplementary material for: Acceptor engineering for NIR-II dyes with high photochemical and biomedical performance
Source: Nat Commun. 2022 Jul 2;13:3815. doi: 10.1038/s41467-022-31521-y (PMC9250501; doi:10.1038/s41467-022-31521-y)
Supplement: Supplementary file 1 — supplementary Information [file 41467_2022_31521_MOESM1_ESM.pdf]

Supplementary Information

Acceptor engineering for NIR-II dyes with high photochemical and biomedical performance

Aiyan Ji[1†], Hongyue Lou[1†], Chunrong Qu[1], Wanglong Lu[1], Yifan Hao[2], Jiafeng Li[1], Yuyang Wu[1,3], Tonghang Chang[1,3], Hao Chen[1,3*], Zhen Cheng[1,3,4*]

1. State Key Laboratory of Drug Research, Molecular Imaging Center, Shanghai Institute of Materia Medica, Chinese Academy of Sciences, Shanghai, 201203, China;
2. Shanghai Institute of Technical Physics of the Chinese Academy of Sciences, Shanghai, 200083, China;
3. University of Chinese Academy of Sciences, No.19A Yuquan Road, Beijing 100049, China;
4. Bohai rim Advanced Research Institute for Drug Discovery, Yantai, 264117, China.

General materials and instruments.

All reactions were performed in glassware containing a Tefloncoated stir bar. All air and moisture sensitive reactions were carried out in flame-dried glassware under a nitrogen atmosphere. Reactive liquid compounds were measured and transferred by gas-tight syringes and were added in the reaction flask through rubber septa. Solvents and chemical reagents were obtained from commercial sources and used without further purifications. Flash column chromatography on silica gel (200-300 mesh) was used for the routine purification of reaction products. The column output was monitored by TLC on silica gel (100-200 mesh) precoated on glass plates (15×50 mm), and spots were visualized by UV light at 254 or 365 nm. ^{1}H and ^{13}C spectra were recorded on Bruker 400 MHz or 500 MHz and the data were recorded using $CDCl_3$ or d_6-DMSO as the solvent. Chemical shifts (δ) are reported in ppm downfield from an internal TMS standard. The NMR spectra analysis processed with MestReNova (Version 12.0.0). Multiplicities are reported as s (singlet), d (doublet), t (triplet), q (quartet), m (multiplet), dd (doublet of doublet), etc. Matrix assisted laser desorption/ionization time of flight mass spectrometry (MALDI-TOF-MS) was performed on Bruker Autoflex Max. Preparative high performance liquid chromatography (HPLC) was performed on a SHMADZU LC-20AR Instrument with PDA detection (column: X-Bridge Protein BEH C4, 5 μm, 250 mm × 10 mm; mobile phase: water/acetonitrile with 0.1 % TFA) (Lab Solutions (Version 1.0.7503. 20679)). UV–vis-NIR absorption spectroscopy were performed on SHIMADZU UV-2600 spectrophotometer (Shimadzu Common

Container (Version 2.70)). Fluorescence emission spectroscopy were performed on iHR320-imaging-spectrometer.

General imaging setup

The NIR-II fluorescence images were taken by vivo imaging system (MARS, Artemis Intelligent Imaging, Shanghai, China), which is equipped with a liquid-nitrogen-cooled InGaAs SWIR camera (NIRvana-640, Teledyne Princeton Instruments, 640 × 512 pixel), SWIR lens with 50 mm focal length (MARS-FAST, Artemis Intelligent Imaging, Shanghai, China) and shadow less illumination using an 808 nm laser (FC-W-808, Changchun New Industries Optoelectronics Technology Co., Ltd.). Another zoom-stereo microscope (Gemini, Artemis Intelligent Imaging, Shanghai, China) equipped with a fixed 1× lens and 0.64×-4.50× zoom lens was also used for large field of view. The fluorescence was then detected by a liquid-nitrogen-cooled InGaAs camera (NIRvana 640, Teledyne Princeton Instruments). The InGaAs camera was cooled to −80°C, the analog to digital conversion rate was set to 2 or 10 MHz, the gain was set to high, and different exposure times were used to achieve sufficient signal. All images were background and blemish corrected within the Light Field imaging software (Version 6.11.4) and processed with image *J* (Version1.8.0). Origin (Version 9.6) was used to analyze the spectrum images. All statistical analyses were performed by Graphpad Prism (Version 8.0.2). Adobe Illustrator CC 2018 (Version 22.1.0) was used to edit the images.

Supplementary Figure 1. Synthetic route for TPA-TQT.

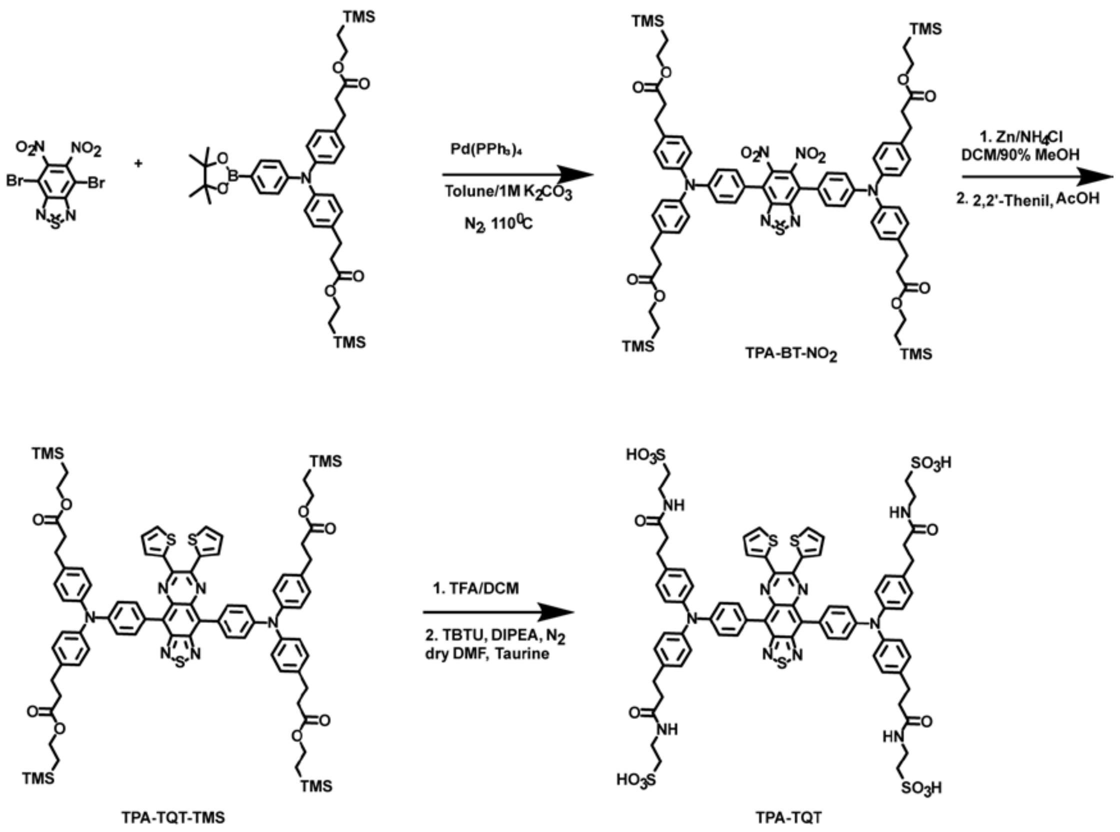

Supplementary Figure 2. Synthetic routes of FT-BBT and FT-TQT.

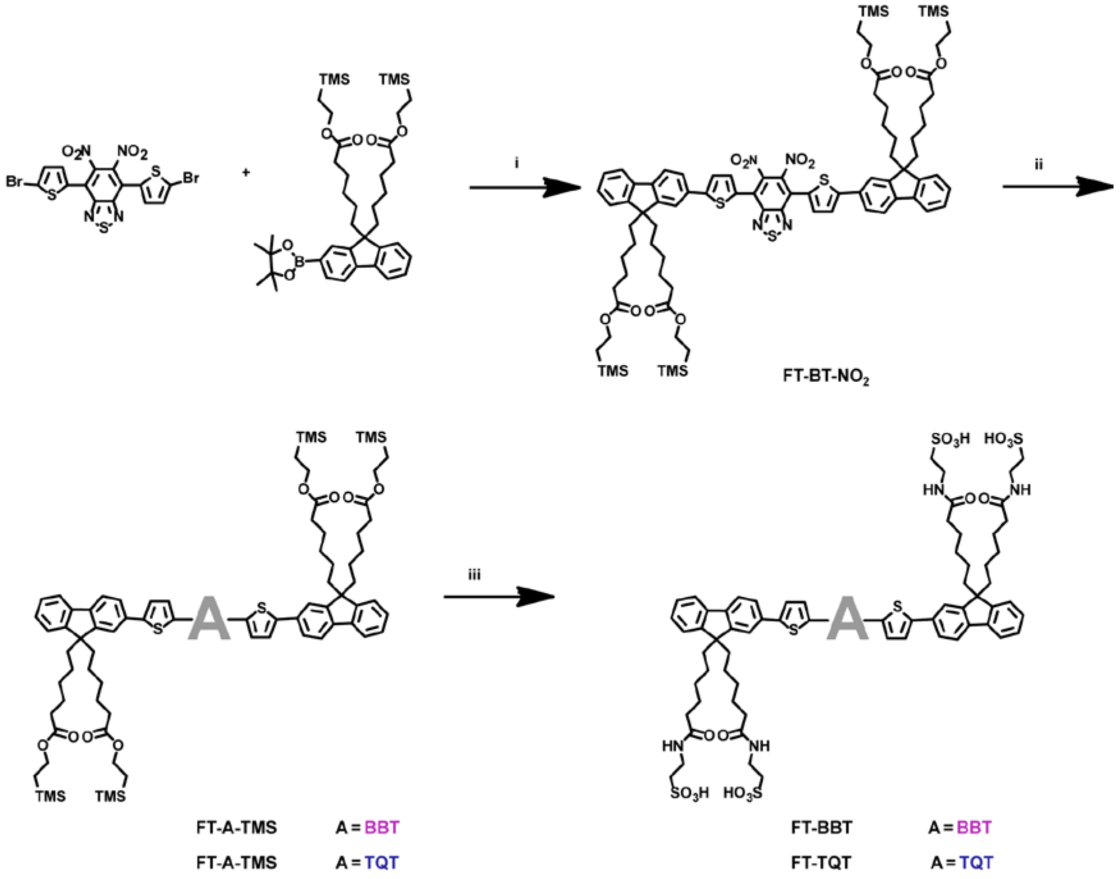

Fluorescent molecules were performed by varying the acceptors. i)**.** Pd(dppf)$_2$Cl$_2$, K$_2$CO$_3$, H$_2$O/toluene, 80°C, N$_2$; ii)**.** 1**.** Zn/NH$_4$Cl, DCM-90%MeOH/H$_2$O, 0°C; 2. pyridine, N-thionylaniline, TMSCl, 80°C (FT-BBT-TMS); AcOH, 2,2′-Thenil, 100°C, N$_2$ (FT-TQT-TMS); iii**).** a. TFA/DCM; b. DIPEA, TBTU, Taurine, Dry DMF, r.t.

Supplementary Figure 3. Highest occupied molecular orbitals (HOMOs) and lowest unoccupied molecular orbitals (LUMOs) of FT-BBT, FT-TQT, and FT-PTQ using Gaussian 03 timedependent density functional theory (TD-DFT) calculations at B3LYP/6-31G(d, p) level; solvent = dichloromethane. To reduce the computational requirements, dialkyl chains were replaced by methyl.

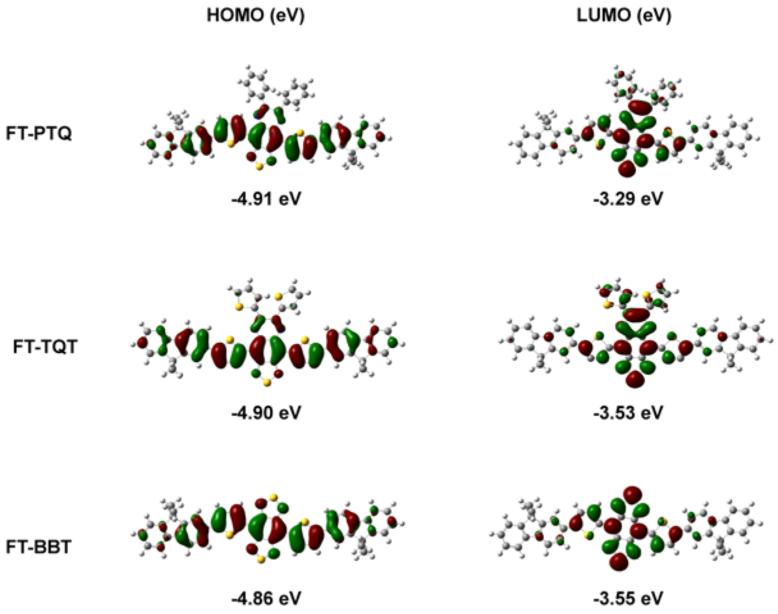

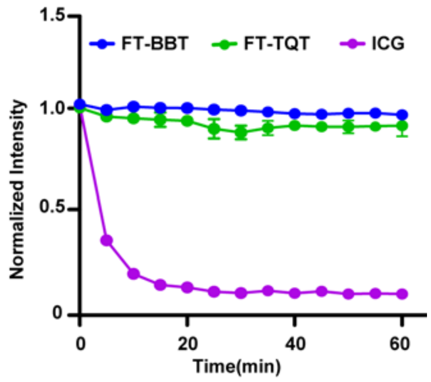

Supplementary Figure 4. Photostability of FTs and ICG under continuous laser irradiation. Data are presented as mean ± s.d. derived from n = 3 independent measurements. (808 nm, 150 mW· cm^{-2})

Supplementary Figure 5. Measurement of the mass extinction coefficient of CH-4T, FT-TQT, and FT-BBT in water (H_2O) at 808 nm. **a, c, e)** Absorption spectra of CH-4T, FT-TQT, and FT-BBT in water with varied concentration. **b, d, f**) Optical density of CH-4T, FT-TQT, and FT-BBT at 808 nm plotted as a function of concentration of CH-4T, FT-TQT, and FT-BBT. The slope was the mass extinction coefficient (3.03×10^3 L M^{-1} cm^{-1}, 1.14×10^4 L M^{-1} cm^{-1}, 8.94×10^3 L M^{-1} cm^{-1} for CH-4T, FT-BBT and FT-TQT).

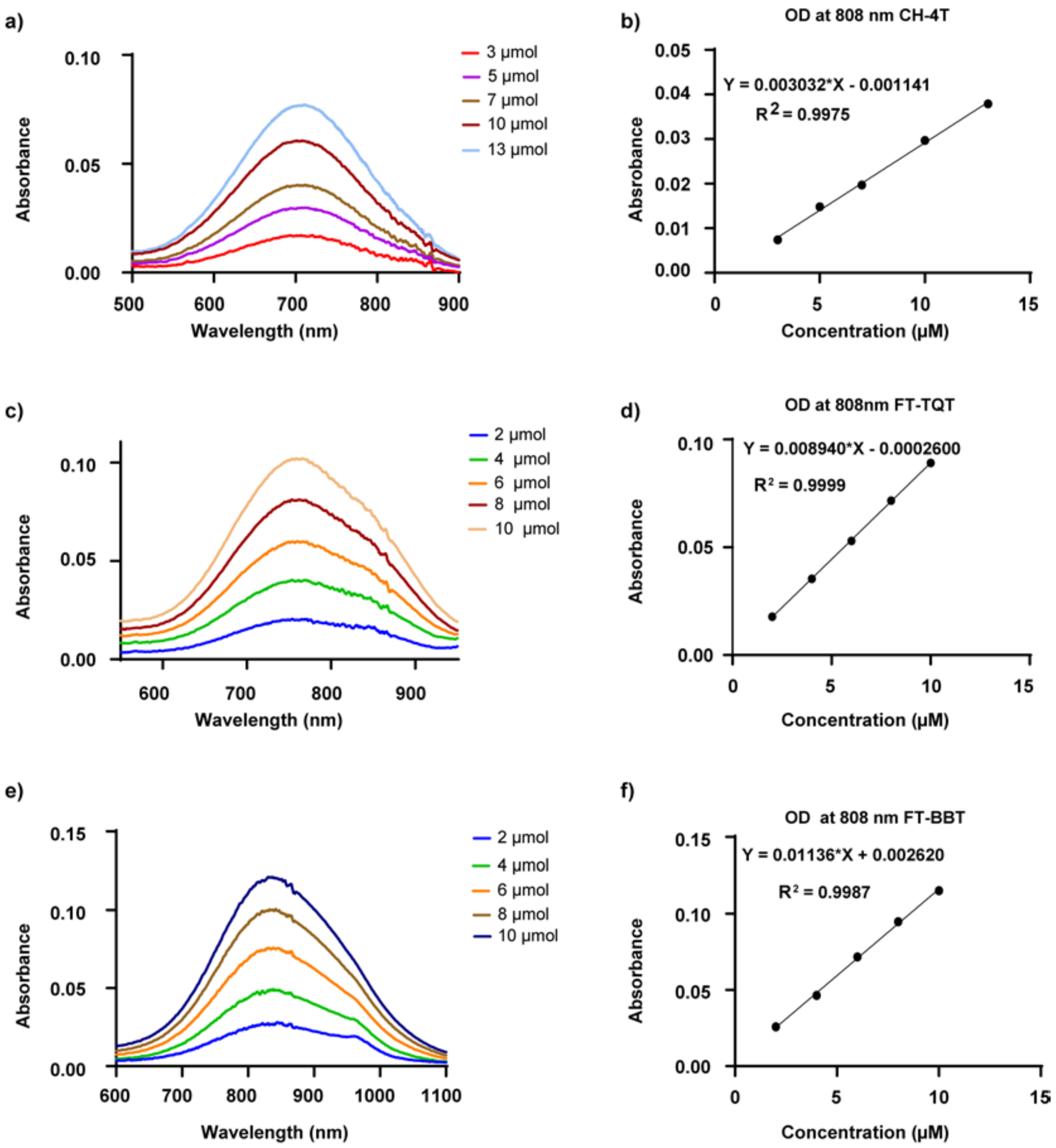

Supplementary Figure 6. Measurement of the mass extinction coefficient of CH-4T, FT-TQT, and FT-BBT in methanol (MeOH) at 808 nm. **a, c, e)** Absorption spectra of CH-4T, FT-TQT, and FT-BBT in MeOH with varied concentration. **b, d, f**) Optical density of CH-4T, FT-TQT, and FT-BBT at 808 nm plotted as a function of concentration of CH-4T, FT-TQT, and FT-BBT. The slope was the mass extinction coefficient (9.88×10^3 L M^{-1} cm^{-1}, 16.25×10^4 L M^{-1} cm^{-1}, 10.66×10^3 L M^{-1} cm^{-1} for CH-4T, FT-BBT and FT-TQT).

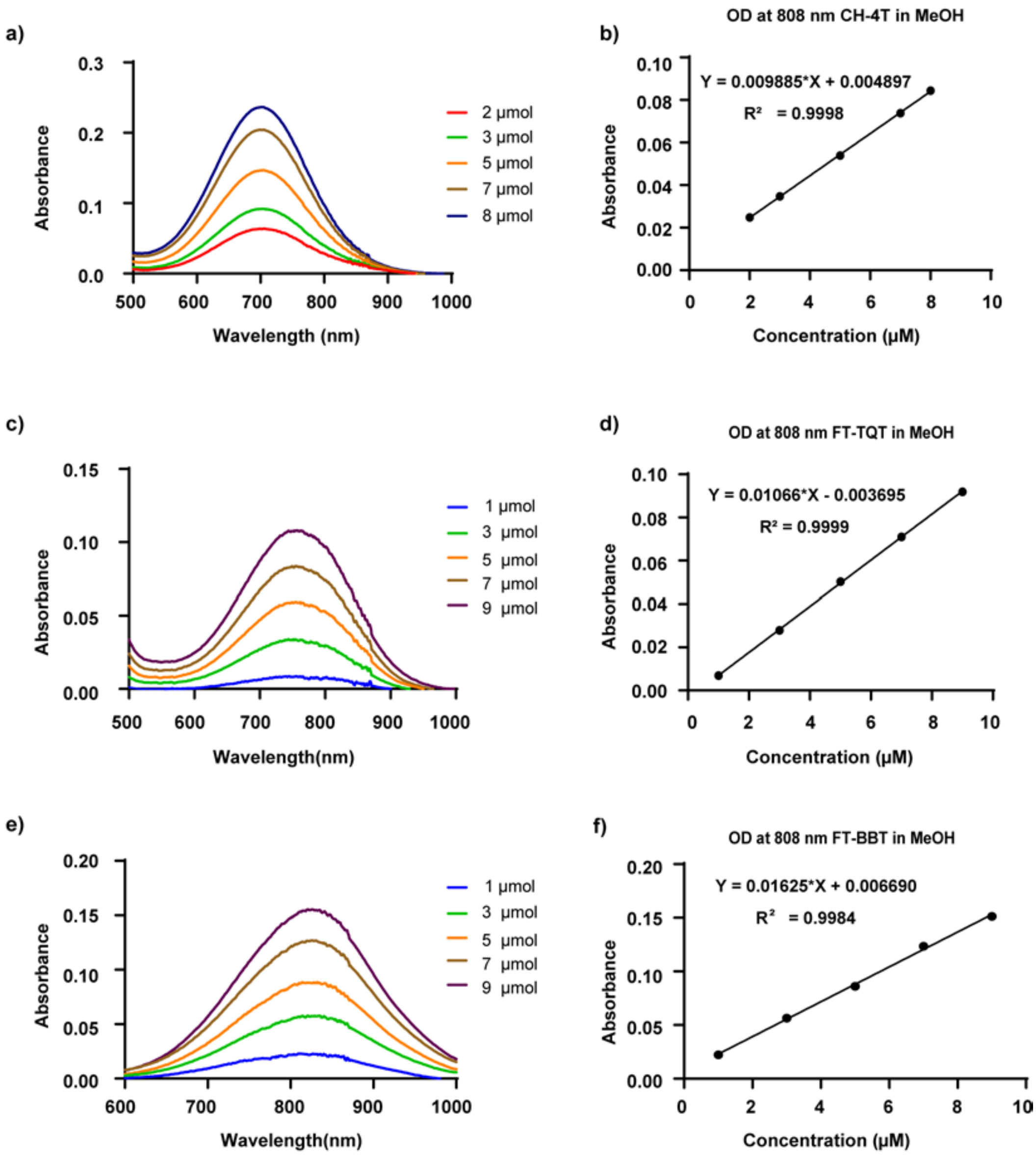

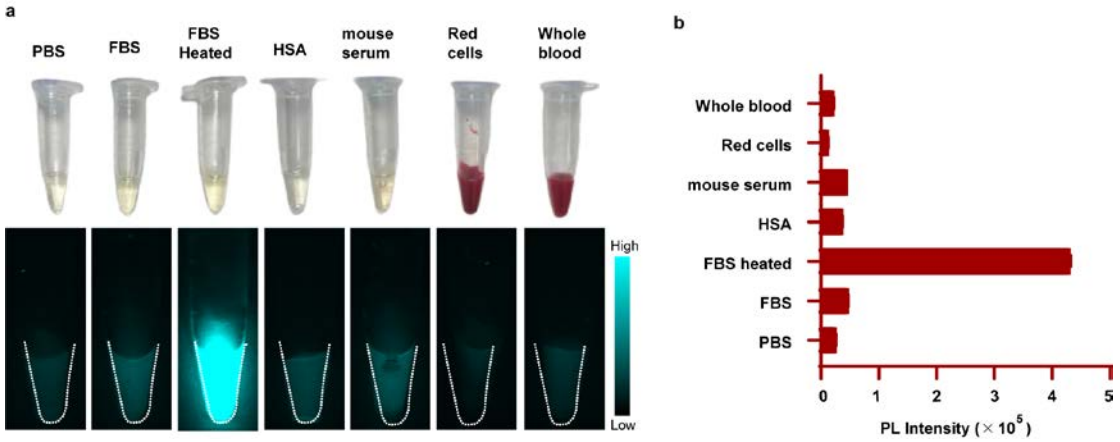

Supplementary Figure 7. Photographs and corresponding NIR-II fluorescent images (10 ms, 1000LP) of FT-TQT in different media. The fluorescence intensity of FT-TQT detected by the InGaAs camera in different media including fresh mice blood, red cells, mouse serum, HSA, FBS and PBS solution. (808 nm, 115 mW cm^{-2}). ([FT-TQT] = 10 μM). HSA: human serum albumin; FBS: fetal bovine serum. For FT-TQT@HSA, the molar ratio of FT-TQT: HSA at 1:1 (HSA: Bidepharm, Lot# BTW600).

Preparation and characterization of FT-TQT@FBS heated complexes.

Briefly, 50 μg FT-TQT was dissolved in 100 μL 50%FBS and vortex the solution to mix evenly. Then the above solution was added in a sealed tube into 70-75^{0}C water baths for 10 min. Finally, the solution was cooled to room temperature for *in vivo* NIR-II imaging.

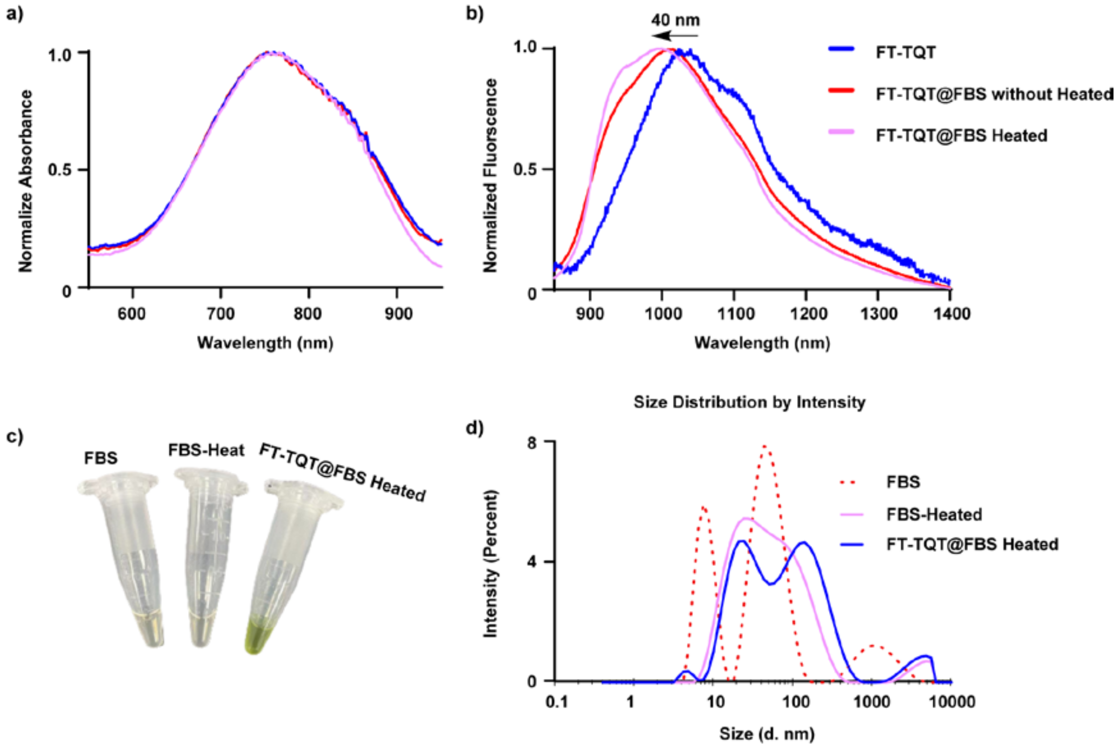

Supplementary Figure 8. **a)** Normalized absorption spectrum of FT-TQT, FT-TQT@FBS without Heated and FT-TQT@FBS Heated in deionized water. **b)** Normalized fluorescence emission spectrum of FT-TQT, FT-TQT@FBS without Heated and FT-TQT@FBS Heated in deionized water. **c)** Photographs of the FBS, FBS-Heated and FT-TQT@FBS Heated in PBS solutions. **d)**. The size of FBS, FBS-Heated and FT-TQT@FBS Heated by dynamic light scattering.

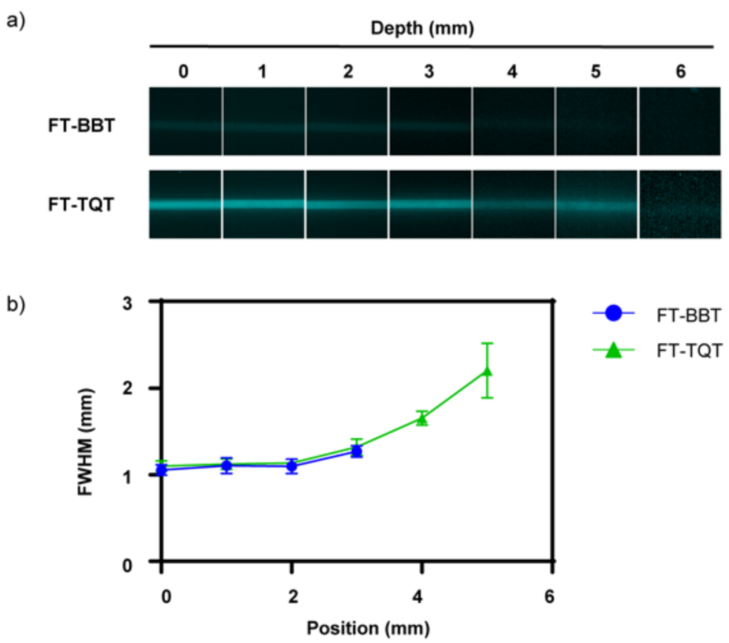

Supplementary Figure 9. Deep penetration of FTs-filled single capillary in 1% Intralipid. **a)** Fluorescence images of capillaries filled with FTs in PBS (pH 7.4), respectively, immersed in 1% Intralipid with varying depth. Imaging signals were collected in 1500 nm region under 808 nm excitation. **b)** Wavelength-dependent full-width at half-maximum (FWHM) of cross-sectional profiles in capillary images as a function of depth. The bars represent mean ± s.d. derived from n = 3 independent measurements.

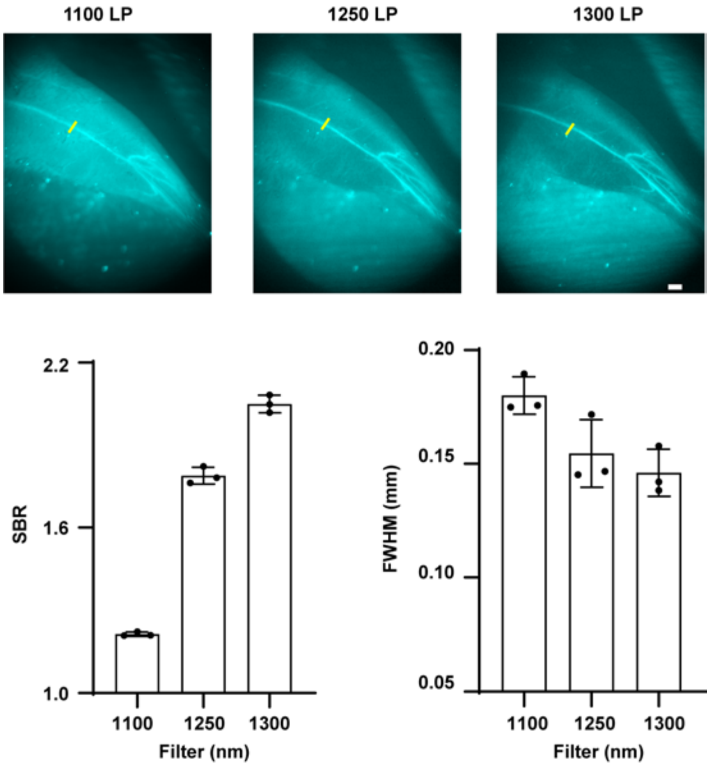

Supplementary Figure 10. NIR bioimaging of FT-BBT injected mice hindlimbs with various long-pass filters. **a)** NIR bioimaging of balb/c mice hindlimb with various long-pass filters by FT-BBT administration (200 μL, 10 mg/kg; 808 nm, 235 mW/cm^{-2}). SBR (**b**) and FWHM. The bars represent mean ± s.d. derived from n = 3 independent measurements. (**c**) of cross-sectional profiles in balb/c mice hindlimb images with various long-pass filters. The bars represent mean ± s.d. derived from n = 3 independent measurements. Scale bar: 1 mm.

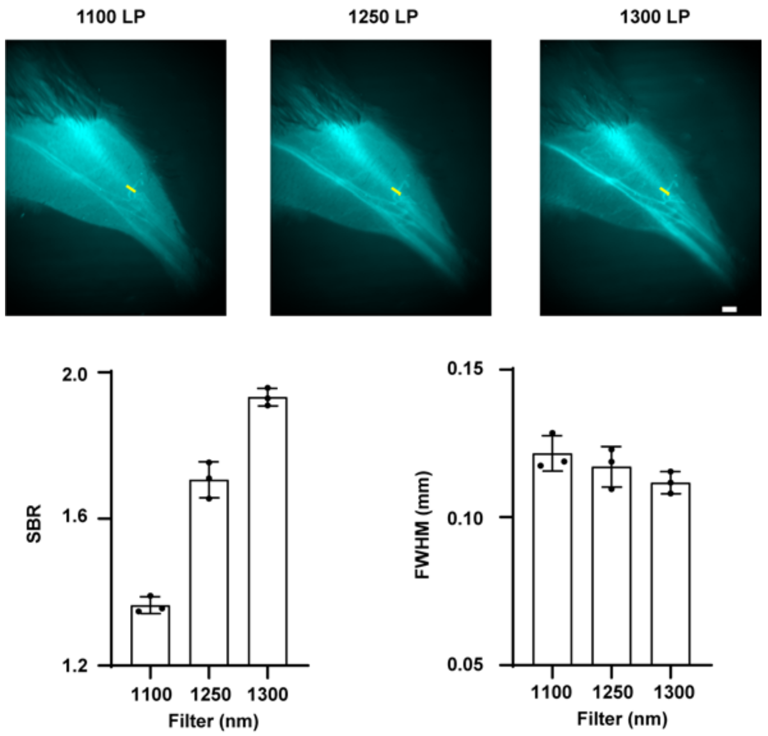

Supplementary Figure 11. NIR bioimaging of FT-TQT injected mice hindlimbs with various long-pass filters. **a)** NIR bioimaging of balb/c mice hindlimb with various long-pass filters by FT-TQT administration (200 µL, 10 mg/kg; 808 nm, 235 mW/cm^{-2}). SBR (**b**) and FWHM. The bars represent mean ± s.d. derived from n = 3 independent measurements. (**c**) of cross-sectional profiles in balb/c mice hindlimb images with various long-pass filters. The bars represent mean ± s.d. derived from n = 3 independent measurements. Scale bar: 1 mm.

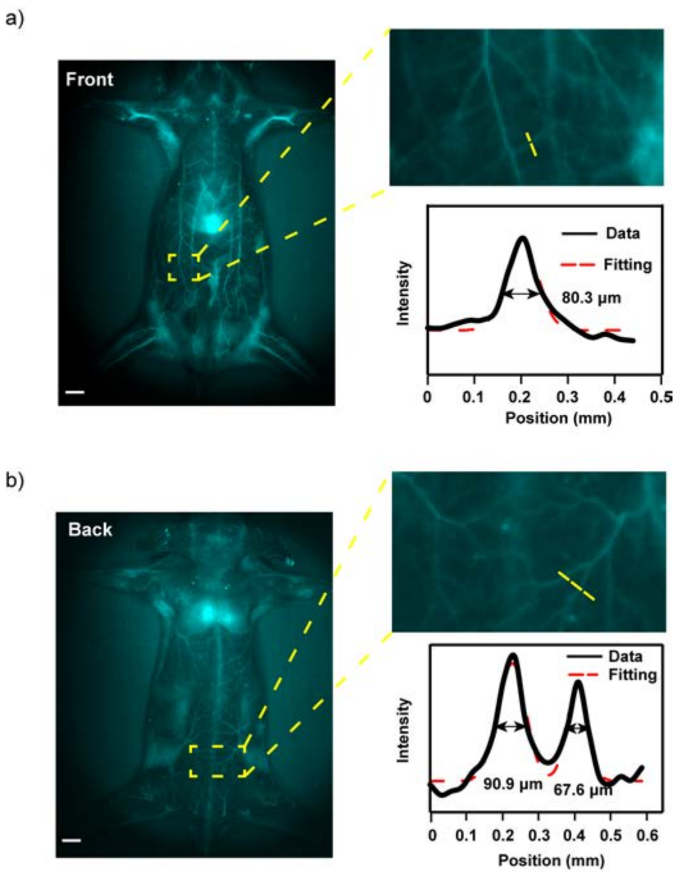

Supplementary Figure 12. Whole body NIR-II fluorescence imaging with FT-TQT at front (**a**) and back (**b**) of mice. The whole body NIR-II fluorescence imaging of mice after i.v. injection of FT-TQT (1400 nm). The measurable vessel exhibited **a** Gaussian-fit diameter of 80.3, 90.9 µm and 67.6 µm on the front and back side, respectively. Scale bars represent 1 mm.

Preliminary cellular toxicity of FT-TQT. Cytotoxicity of FT-TQT was evaluated by using MTT (3-(4,5- dimethylthiazol-2-yl)- 2,5-diphenyl tetrazolium bromide) assay. Murine fibroblast 3T3 cells and Human osteosarcoma 143B cells were plated into a 96 well plate at a concentration of 1 x 10^4 and 5 x 10^3 cells/well in Dulbecco's modified Eagle medium (DMEM) with 10% FBS at 37°C and 5% CO_2, and 24 h later, the cells were incubated with FT-TQT with different concentrations (100 μM, 75 μM, 50 μM, 25 μM, 10 μM, 0 μM) for 24 h. Then 20 μL of the 5 mg/mL MTT stock solution was added and incubated for 4 hours. After removing medium, 150 μL of dimethylsulfoxide was added to dissolve the formazan crystals precipitates. After shaking the cell plate for 5 min, the absorbance (A) at a wavelength of 490 nm was measured with a Tecan microplate reader (SoftMax Pro 7.1.lnk (Version 7.1.1)). The normalized cell viability was calculated by (A4T/Ablank), (Y values). The concentrations of FT-TQT were calculated in log scales (X values). Mean value and standard deviation were calculated using GraphPad Prism (Version 8.0.2). Dose-response data table was chosen, with nonlinear regression the curve was obtained. Error bars represent standard deviation. All samples were done in triplicate and the experiment was replicated five times.

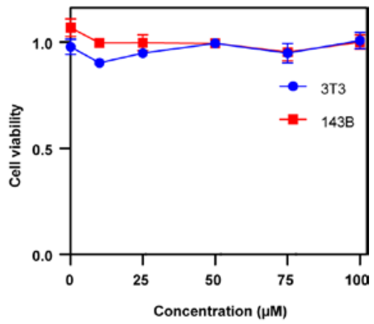

Supplementary Figure 13. Cell viability of 3T3 (blue) and 143B (red) treated with FT-TQT at different concentrations ranging from 0 to 100 μM for 24 h. Data are

presented as mean ± s.d. derived from n = 5 independent measurements.

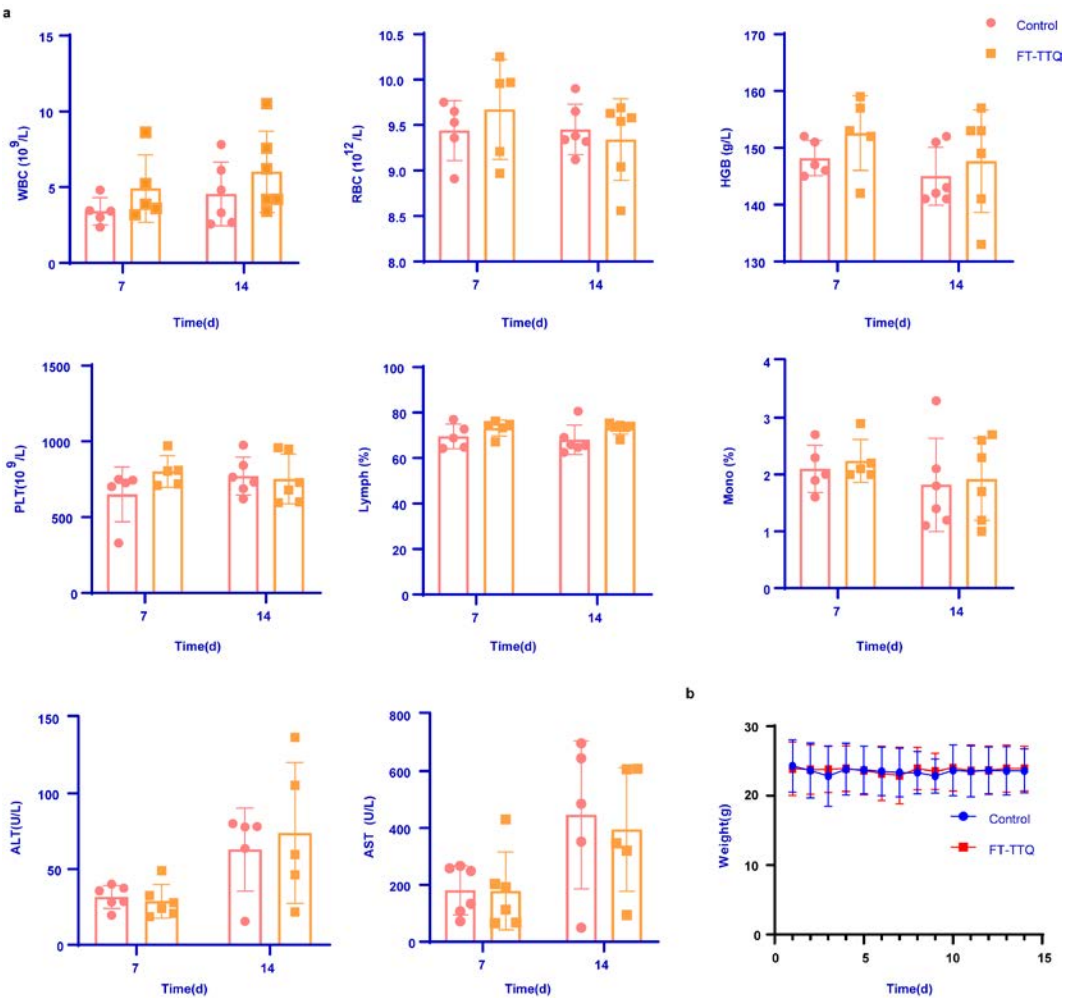

Supplementary Figure 14. *In vivo* biotoxicity of FT-TQT. **a**) Routine blood test and blood biochemistry test results of samples collected from the mice injected with 1×PBS and 5 mg/kg of FT-TQT (200 µL), respectively. The blood samples were collected and analyzed 7 days and 14 days post different treatments (n = 6, 3♂3♀). The bars represent mean ± s.d. derived from n ≥ 5 independent animals (Unqualified blood samples were excluded). **b**) The weight of FT-TQT treated mice were not significantly different from the control in the 14 days. The bars represent mean ± s.d. derived from n = 6 independent animals. Abbreviations: white blood cell, WBC; red blood cell, RBC; blood platelet, PLT; hemoglobin, HGB; lymphocyte, Lymph; monocyte, Mono; alanine aminotransferase, ALT; aspartate transaminase, AST.

Supplementary Figure 15: *Ex Vivo* Biodistribution of FT-TQT. *Ex vivo* biodistribution studies were further performed at 7 and 14 days post-injection of FT-TQT (100 µg) through tail vein to evaluate its distribution in vital organs including heart, liver, spleen, lung, and kidney. The organs were harvested and imaged under NIR-II imaging at 1000 nm LP filter with 30 ms exposure time. After NIR-II imaging, the organs were fixed in 4% paraformaldehyde for 48 h, and embedded with paraffin. Sections (100 µm) were stained with H&E.

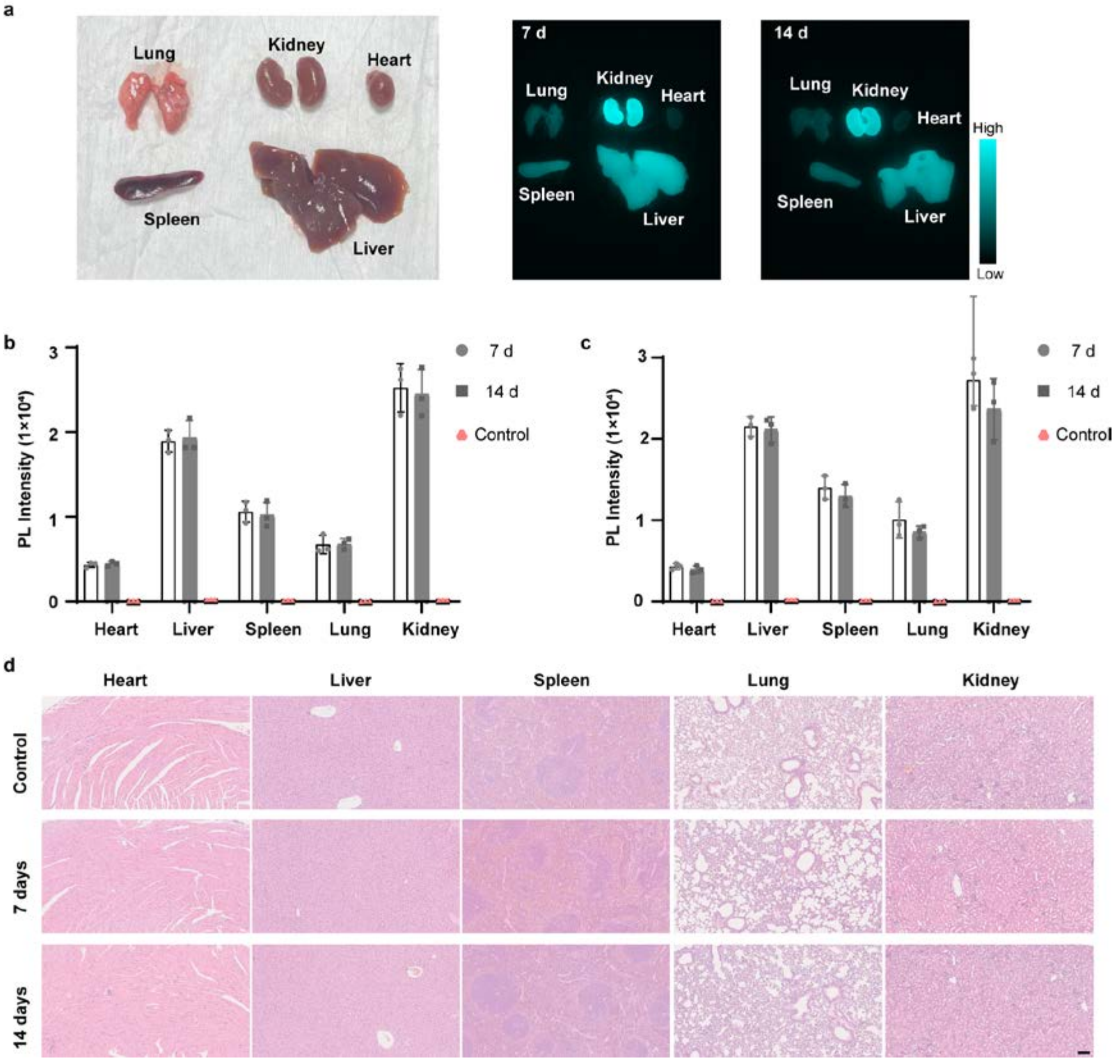

Toxicity and biodistribution assay. **a)** *Ex vivo* NIR-II imaging of the vital organs at 7 and 14 days post-injection. Histogram of the fluorescence intensities of the organs and

tissues before **(b)** and after **(c)** homogenizing. Data as mean values ± s.d. derived from n = 3 independent animals. **d)** Representative major organ histology (H&E stained) of FT-TQT treated mice at 7 and 14 days post-injection. The results derived from n = 3 independent measurements. PL Intensity: photoluminescence intensity. Scale bar: 100 µm.

Supplementary Figure 16: *Ex Vivo* Biodistribution of FT-TQT@FBS.

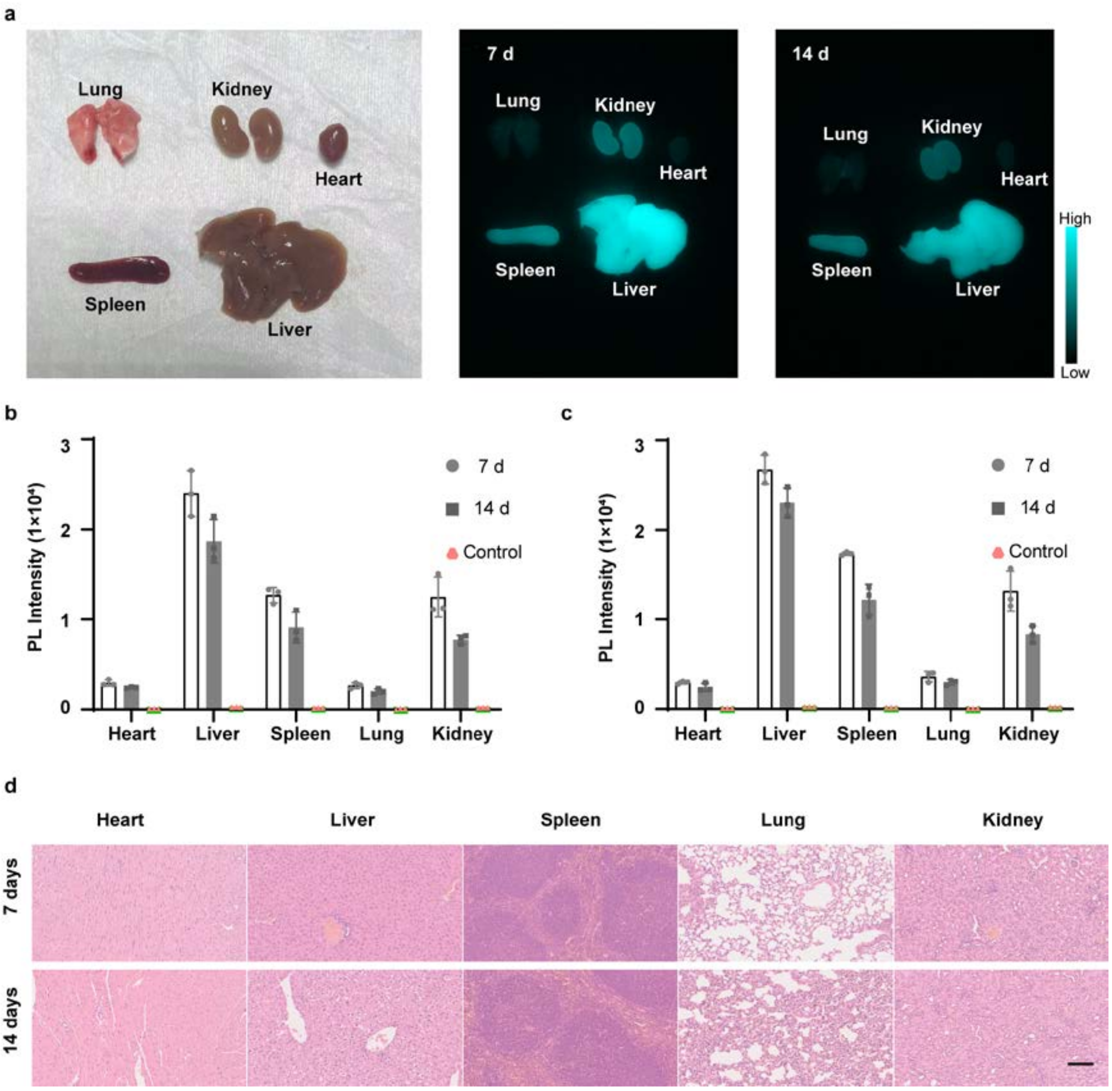

Toxicity and biodistribution assay of FT-TQT@FBS. **a)** *Ex vivo* NIR-II imaging of the vital organs at 7 and 14 days post-injection. Histogram of the fluorescence intensities of the organs and tissues before **(b)** and after **(c)** homogenizing. Data as mean values ± s.d. derived from n = 3 independent mice. **d)** Representative major organ histology (H&E stained) of FT-TQT@FBS treated mice at 7 and 14 days post-injection. The results derived from n = 3 independent measurements. PL Intensity: photoluminescence intensity. Scale bar: 100 µm. (Laser, 808 nm; Power, 90 mW·cm^{-2}; Exposure time, 30 ms; 1000 nm Long Pass filter)

Supplementary Figure 17. The FT-TQT concentration curve in blood. The Balb/c mice (n = 3) were injected FT-TQT (4 mg/kg)，and the blood was collected in capillarity tubes at different time points. The average fluorescence measured with InGaAs camera at 1000 nm filter, 808 nm excitation, and exposure time of 300 ms. The concentration was calculated by linearity fluorescence emission curves of diluted injected solution. The bars represent mean ± s.d. derived from n = 3 independent mice.

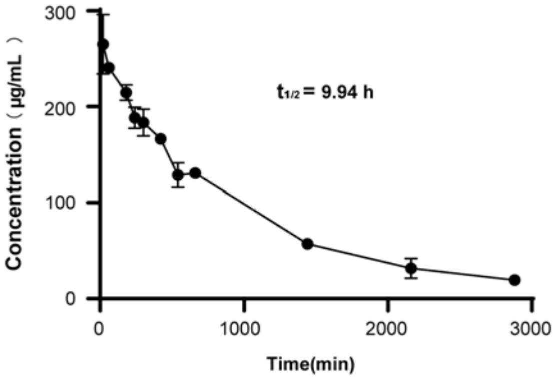

Parameter	Unit	Value
t1/2	h	9.94027793
V	(mg)/(μg/mL)	3.01321712
CL	(mg)/(μg/mL)/h	0.21011515
AUC 0-t	μg/mL*h	3788.26203
AUC 0-inf	μg/mL*h	3926.41851
AUMC	μg/mL*h^2	56307.942
MRT	h	14.3407897
Vss	mg/(μg/mL)	3.01321712

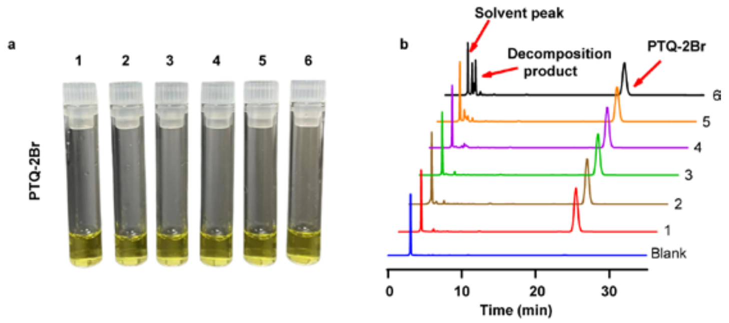

Supplementary Figure 18. The stability of PTQ-2Br at various acid-base conditions. **a)**. Bright field images of PTQ-2Br in MeOH (5%DMF, 0.1 mg/mL) at various acid-base conditions. 1: control solution; 2: control solution + excess trifluoroacetic acid (TFA); 3: control solution + 1μL Triethylamine (TEA); 4: control solution + 5 μL Triethylamine (TEA); 5: control solution + 10 μL Triethylamine (TEA); 6: control solution + 20 μL Triethylamine (TEA). **b).** HPLC chromatograms of PTQ-2Br at various acid-base conditions. With the increase of alkali concentration, the PTQ-2Br's peak area decreased.

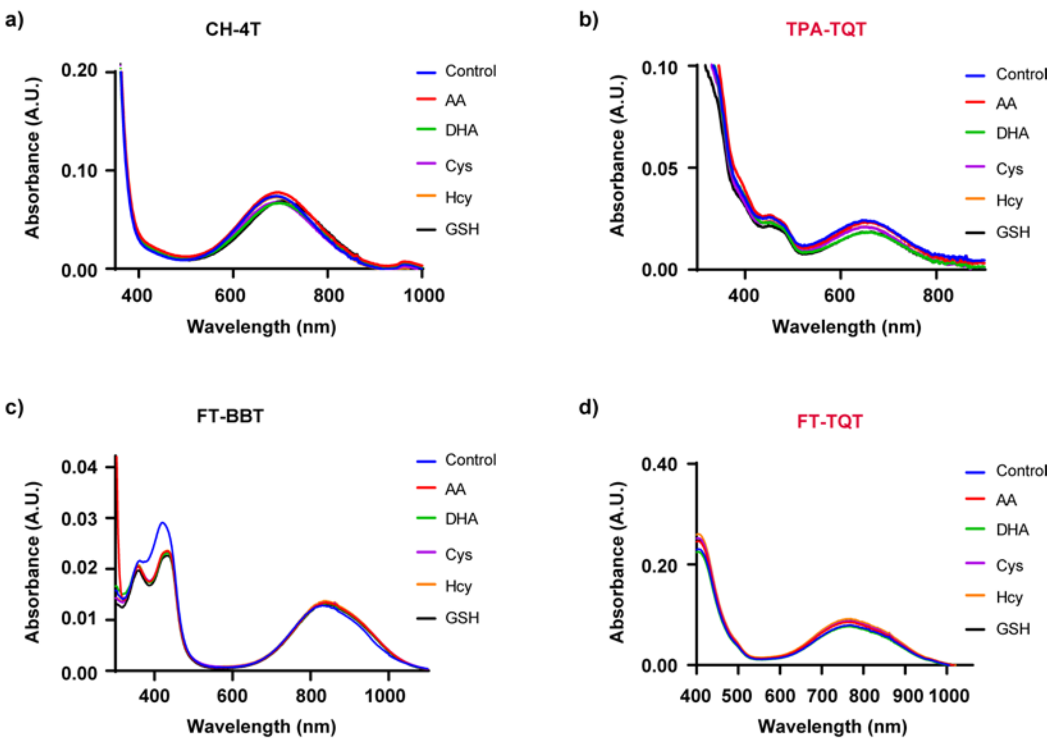

Supplementary Figure 19. Changes of absorption curves of dyes (10 μM), CH-4T **(a)**, TPA-TQT **(b)**, FT-BBT **(c),** and FT-TQT **(d)** after reaction with active biomolecules. ascorbic acid, AA; dehydroascorbic acid, DHA; L-Cysteine, Cys; Glutathione, GSH; DL-Homocysteine, Hcy. Incubation conditions: 37^0C water bath for one hour. A.U. means Absorbance Unit.

Preparation of Reactive Oxygen/Nitrogen Species (ROS/RNS).

Nitrite (NO_2^-) was prepared by dissolving sodium nitrite in deionized water. Hypochloride anion (ClO^-) was prepared by NaClO. H_2O_2 solution (10 mM) was provided by diluting 30% H_2O_2. Peroxynitrite ($ONOO^-$) solution was generated by H_2O_2 solution (10 mM) reacting with $NaNO_2$ (10 mM). The single oxygen ($^1O_2$) was produced by reaction between NaClO (10 mM) and H_2O_2 (10 mM). CH-4T, TPA-TQT, FT-BBT, and FT-TQT (10 μM) were incubated with these active molecules (1 mM) in a 37^0C water bath for one hour. Then, cooling to room temperature and measure their absorption.

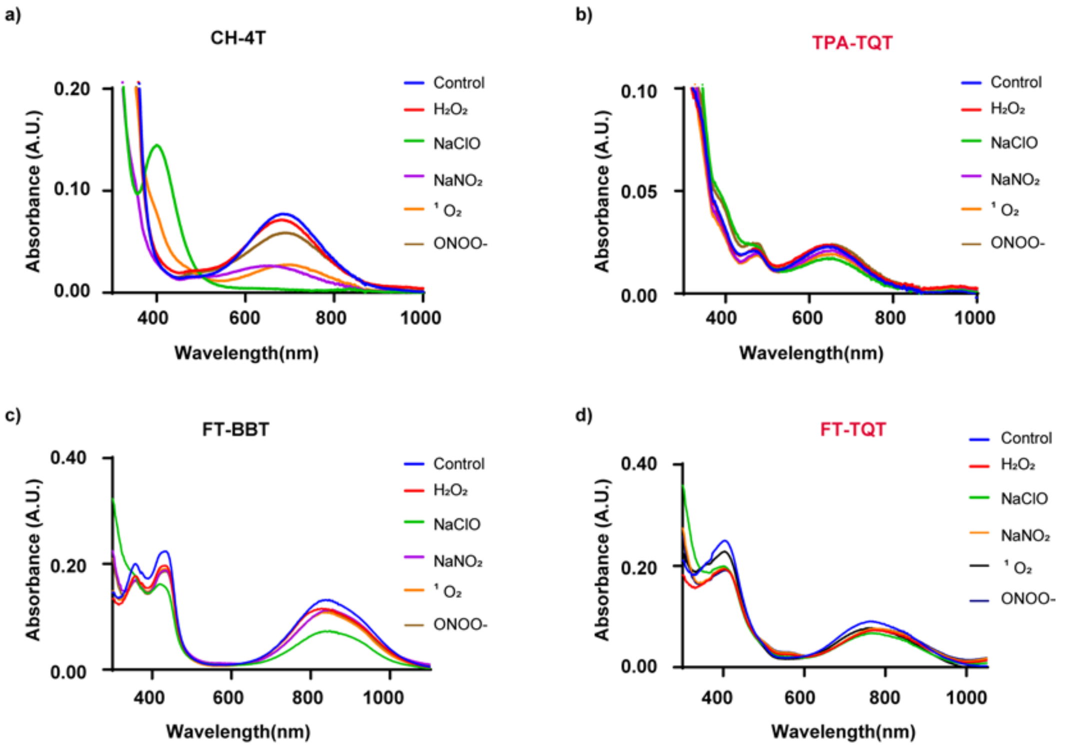

Supplementary Figure 20. Changes of absorption curves of CH-4T **(a)**, TPA-TQT **(b)**, FT-BBT **(c),** and FT-TQT **(d)** after reaction with ROS/RNS. A.U. means Absorbance Unit.

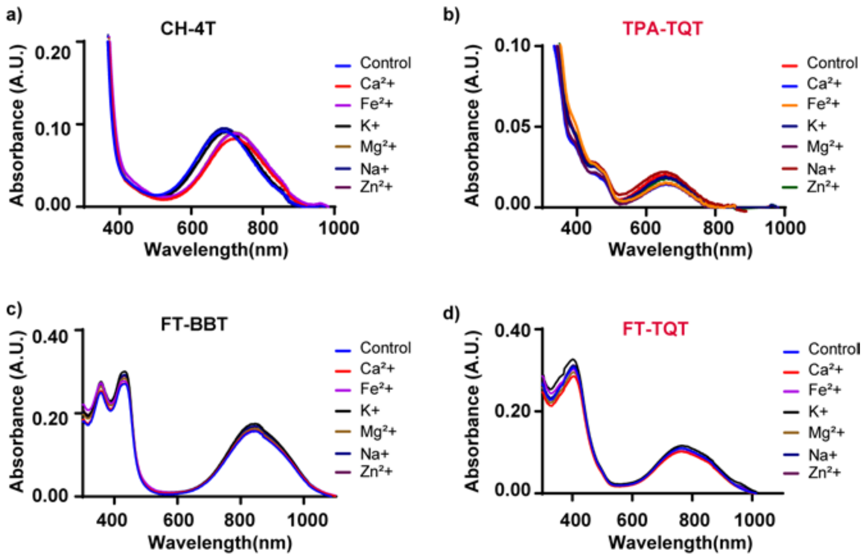

Supplementary Figure 21. Changes of absorption curves of dyes (10 μM), CH-4T **(a)**, TPA-TQT **(b)**, FT-BBT **(c),** and FT-TQT **(d)** after reaction with metal ions (1 mM). Incubation conditions: 37^{0}C water bath for one hour. A.U. means Absorbance Unit.

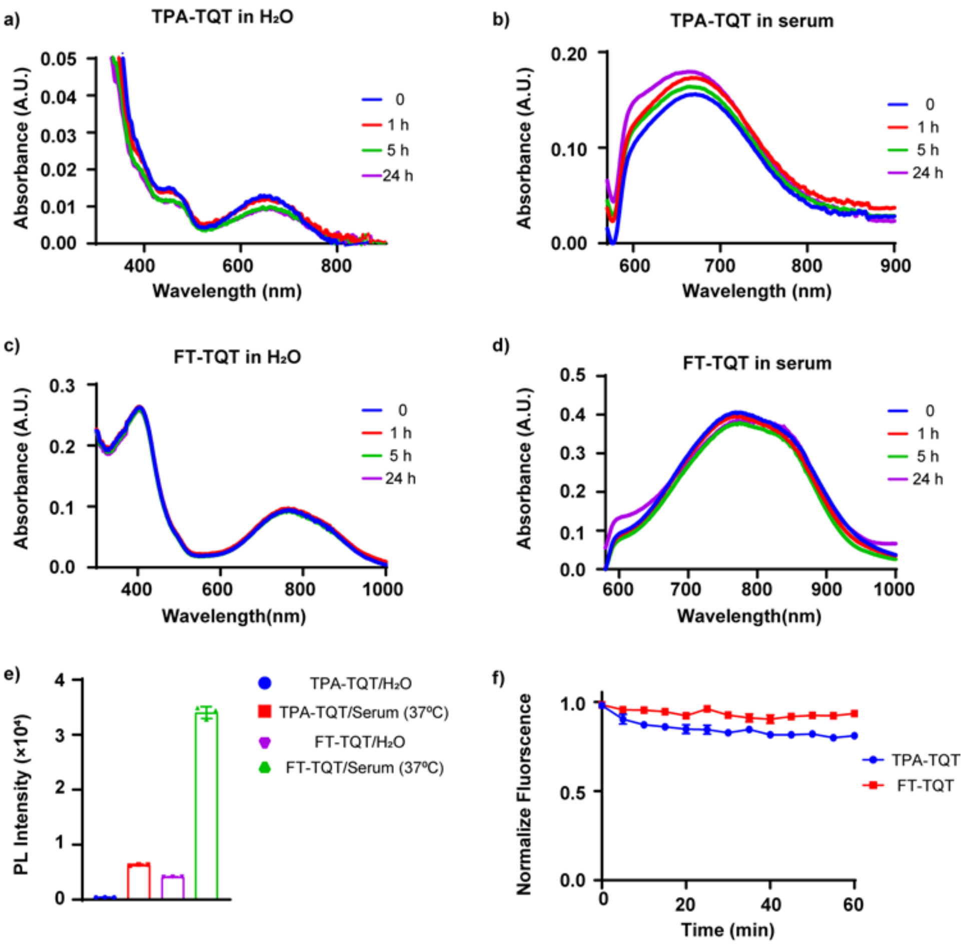

Supplementary Figure 22. The stability of TPA-TQT and FT-TQT in mouse serum. The absorption spectra of TPA-TQT in H_2O (**a**) and mouse serum **(b)** at different times**;** The absorption spectra of FT-TQT in H_2O **(c)** and mouse serum **(d)** at different times; **e)** The fluorescence intensity of TPA-TQT and FT-TQT with the same concentration (8.5 μM) in water and mouse serum at 37℃ for 1 hour. Data are presented as mean ± s.d. derived from n = 3 independent measurements. (1000 LP, 100 ms, 100 mW· cm^{-2}); **f)** Photostability of TPA-TQT and FT-TQT in serum under continuous laser irradiation (808 nm, 100 mW· cm^{-2}). A.U. means Absorbance Unit.

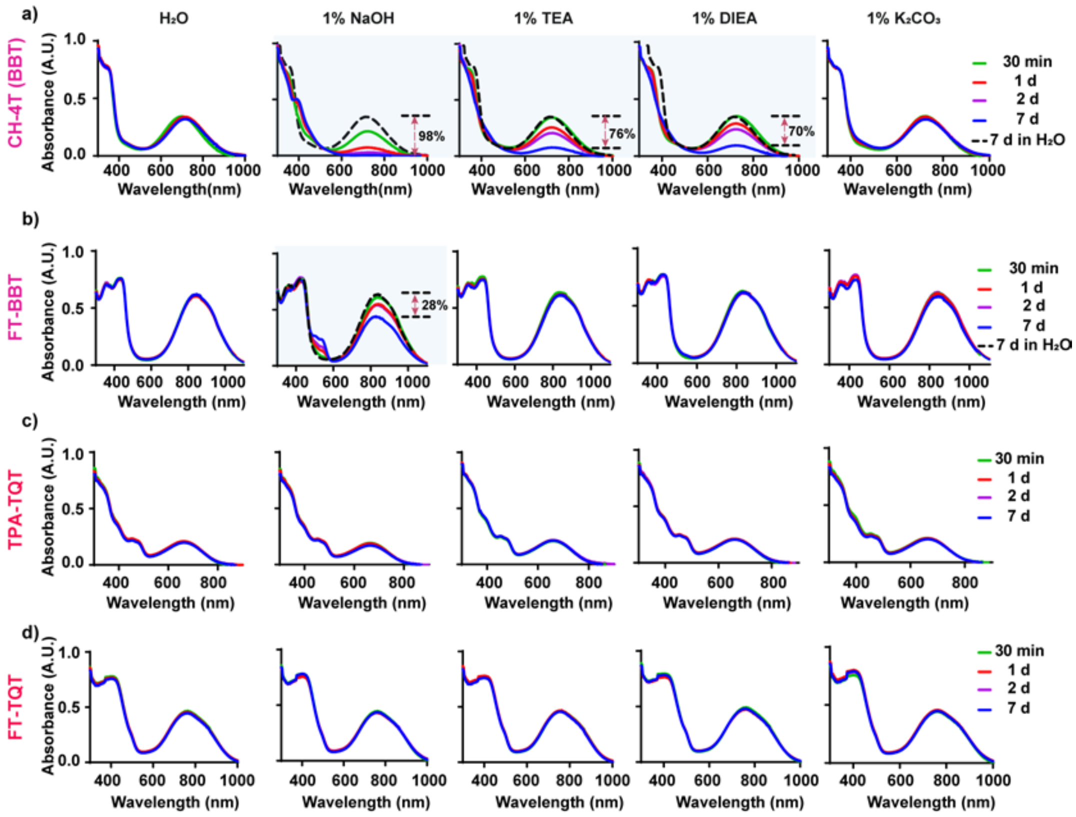

Supplementary Figure 23. The stability of CH-4T **(a)**, FT-BBT **(b)**, TPA-TQT **(c),** and FT-TQT **(d)** in different alkaline aqueous solutions at room temperature. Dye concentration: 50 µM; 1% represents mass concentration (g/100 mL or mL/100 mL). Sodium hydroxide, NaOH; Triethylamine, TEA; N, N-Diisopropylethylamine, DIPEA; Potassium carbonate, K_2CO_3. A.U. means Absorbance Unit.

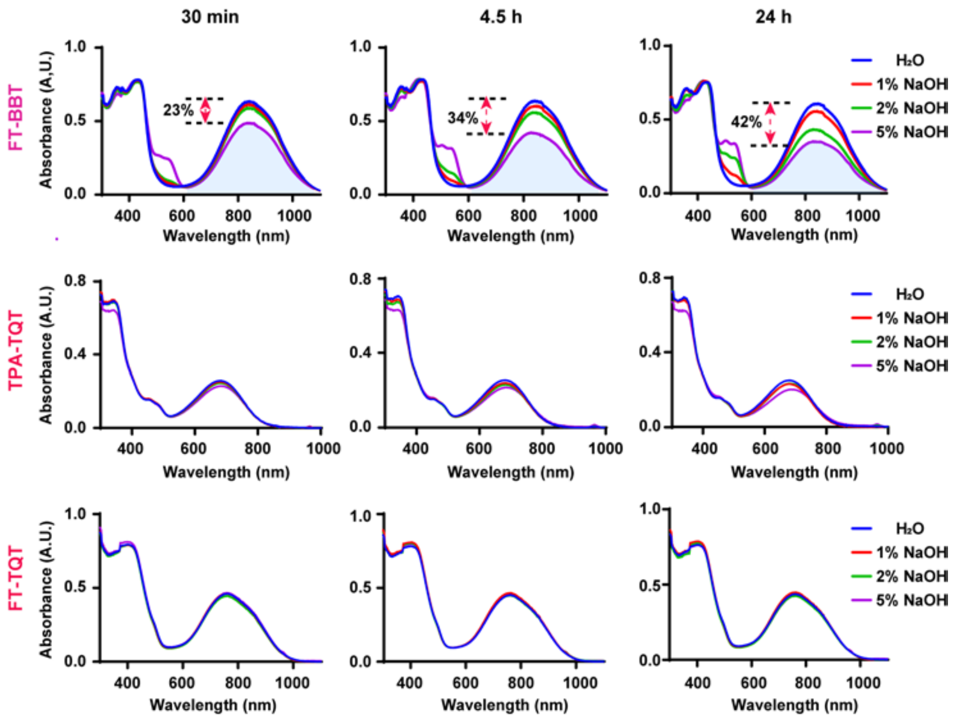

Supplementary Figure 24. The stability of FT-BBT, TPA-TQT, and FT-TQT in different mass concentrations of NaOH at room temperature for different times. Dye concentration: 50 µM. A.U. means Absorbance Unit.

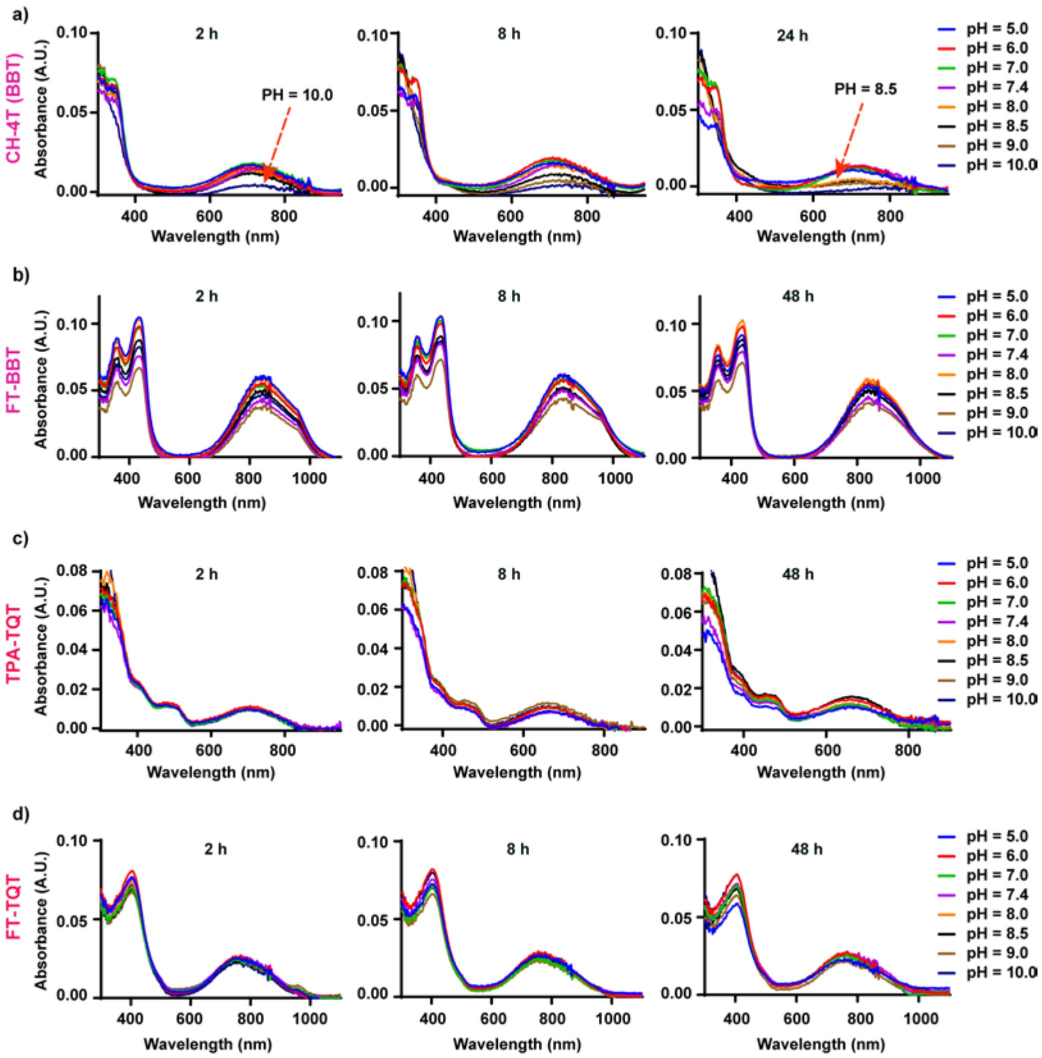

Supplementary Figure 25. The stability of CH-4T **(a),** FT-BBT **(b),** TPA-TQT **(c),** and FT-TQT **(d)** under different pH conditions. Incubation conditions: 37^{0}C water bath. [CH-4T, FT-BBT, FT-TQT]: 2 µM; [TPA-TQT]: 6 µM. A.U. means Absorbance Unit.

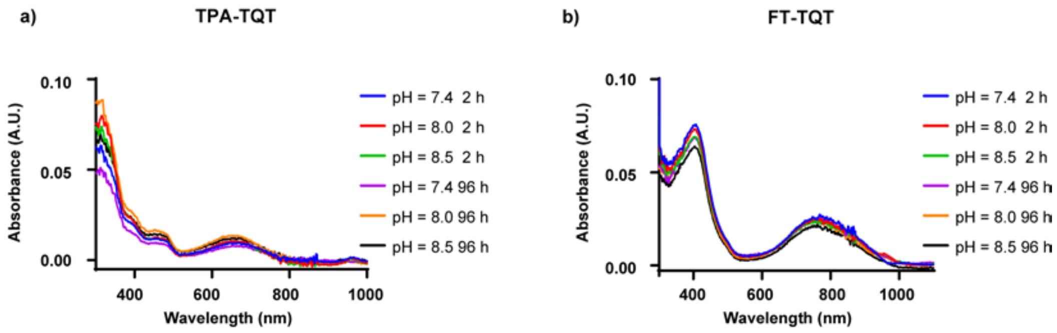

Supplementary Figure 26. The stability of TPA-TQT **(a)** and FT-TQT **(b)** in pH 8.0, 8.5 for 96 h. [TPA-TQT]:6 μM; [FT-TQT]:2 μM. A.U. means Absorbance Unit.

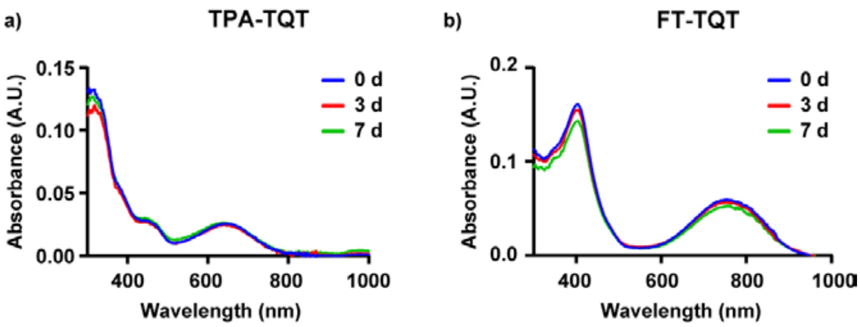

Supplementary Figure 27. The stability of TPA-TQT and FT-TQT in MeOH at different time points. [TPA-TQT]:10 μM; [FT-TQT]:5 μM. A.U. means Absorbance Unit.

Vessel Density Characterization

The vascular quantification algorithm is based on a modified Hessian matrix method [1,2].

Step 1, Enhance high-frequency direction information. First, before computing the Hessian matrix of the image, the direction filters was used to acquire high-frequency direction information of the image. The definition of the direction operators are as follows:

$$g1 = \begin{bmatrix} -0.5 & 1 & -0.5 \\ -0.5 & 1 & -0.5 \\ -0.5 & 1 & -0.5 \end{bmatrix}$$

$$g2 = \begin{bmatrix} -0.5 & -0.5 & -0.5 \\ 1 & 1 & 1 \\ -0.5 & -0.5 & -0.5 \end{bmatrix}$$

$$g3 = \begin{bmatrix} 1 & -0.5 & -0.5 \\ -0.5 & 1 & -0.5 \\ -0.5 & -0.5 & 1 \end{bmatrix}$$

$$g4 = \begin{bmatrix} -0.5 & -0.5 & 1 \\ -0.5 & 1 & -0.5 \\ 1 & -0.5 & -0.5 \end{bmatrix}$$

$g1, g2, g3, g4$ are four direction operators. And the original image is convolved with the direction operator to obtain the information of the corresponding direction. Then we choose the max value as the direction message of original image. The definition of enhancement of image is as follow:

$$I_{en} = normalized(I + e \times \max(I * g_n)), n = 1,2,3,4 \quad (1)$$

Normalized stands for that each element in the matrix is divided by the maximum value of the matrix. * stands for convolution. I means original image. And I_{en}

stands for result of enhance. e stands amplification factor.

Step 2. Compute the Hessian matrix of the image. The Hessian matrix of image $I_{en}(x, y)$ under scale s which essentially extracts the local curvature of the structure was computed as

$$H = \begin{bmatrix} \frac{\partial^2 I_{en}(x,y,s)}{\partial x^2} & \frac{\partial^2 I_{en}(x,y,s)}{\partial x \partial y} \\ \frac{\partial^2 I_{en}(x,y,s)}{\partial y \partial x} & \frac{\partial^2 I_{en}(x,y,s)}{\partial y^2} \end{bmatrix} \quad (2)$$

where $I_{en}(x, y, s)$ is defined as the convolution of $I_{en}(x, y)$ and a smoothing Gaussian kernel $G(x, y, s)$,with s controls the size of the kernel.

Step 3. Get the vessel map of images. The eigenvalues of the Hessian matrix were computed, which represent the curvature along the principal directions corresponding to respective eigenvectors. Since blood vessels in images are shown as bright tubular structures[1]. So, the eigenvalue with smaller absolute value, which represents the curvature along the direction of a vessel should be close to zeros. And the one with bigger absolute value which represents the curvature along the perpendicular direction of the vessel[2]. We used limitation factors to extract vessels and suppress noise as follows:

$$f(s) = \begin{Bmatrix} 0, if\, \lambda_2 > 0 \\ 0, if\, |\lambda_1| < \varepsilon = 0.8 \\ \lambda_2, else \end{Bmatrix} \quad (3)$$

where λ_1, λ_2 stand for the eigenvalue with smaller absolute value and the eigenvalue with bigger absolute value respectively. And s stands for the scale factor which takes a positive integer between 1 and 6 in this study. ε is a small number which is equal to 0.8.

Finally, the vessel map of images can be obtained as:

$$f = \max | f(s) |, s = 1,2,3,4,5,6 \quad (4)$$

Step 4. Use the mean value of f to characterize blood vessel density. The more blood vessels extracted from the image made the more non-zero items in vessel map f. The clearer the blood vessels made the larger the non-zero items in f. Therefore, the mean value of f is positively correlated with blood vessel density. Here we used it to represent the vessel density approximately.

$$d = average(f) \quad (5)$$

Where d means the vessel density. $average$ stands for the average of f.

Supplementary Table 1: Quantum yield of FT-BBT, FT-TQT and FT-TQT@FBS complexes**.** In order to measure the quantum yield of FT-BBT, FT-TQT and FT-TQT@FBS complexes**,** a reference fluorophore emitting in the NIR-II was chosen. While there is some debate over the true quantum yield of IR-26, 0.5% is the accepted value. The quantum yield was calculated in the following manner:

$$\mathrm{QY} = QY_{ref} \times \frac{n^2}{n_{ref}^2}\left(\frac{A_{ref}}{I_{ref}}\right)\frac{I_{sample}}{A_{sample}}$$

Note: QY_{ref} of IR-26 in dichloroethane is 0.5%; the refractive index (n_{H2O}, n_{MeOH} and n_{ref}) are 1.333, 1.329 and 1.414.

Instead of comparing the integrated fluorescence intensity at a single concentration to that of the reference, 5 difference concentrations at or below OD 0.1 were measured and the integrated fluorescence was plotted against absorbance for IR-26, FT-BBT, FT-TQT and FT-TQT@FBS complexes. Comparison of the slopes led to the determination of the quantum yield of FT-BBT, FT-TQT and FT-TQT@FBS complexes.

Supplementary Table 1. Quantum yield and brightness values of FT-BBT, FT-TQT, and FT-TQT@FBS complexes in MeOH and H_2O.

Fluorophore	QY_{MeOH} (%)	QY_{H2O} (%)	ε_{H2O} ($x10^3 M^{-1} \cdot cm^{-1}$)	ε_{MeOH} ($x10^3 \cdot M^{-1} \cdot cm^{-1}$)	$\varepsilon * \Phi_{H2O}$ ($M^{-1} \cdot cm^{-1}$)	$\varepsilon * \Phi_{MeOH}$ ($M^{-1} \cdot cm^{-1}$)
**FT-BBT**	0.23	---	11.4	16.25	---	37.4.
**FT-TQT**	0.49	0.025	8.94	10.66	2.24	52.2
**CH-4T**	0.11	0.06	3.03	9.88	1.82	10.87
**FTTQT@FBS**	---	0.20	---		---	---
**IR-26(DCE)**	0.05					

Supplementary Table 2. The summary of photophysical properties of reported NIR-II water-soluble fluorescent dyes.

Probe	Absorbance (nm)	Emission (nm)	Φ (IR-26 = 0.5%)	Acceptor	Reference
CH1055-PEG	755	1055	0.3%	BBT	Nat. Mater. 2016, 15, 235-242.
CH-4T	~ 738	~ 1055	0.098%	BBT	Nat. Commun. 2017, 8, 15269.
IR-FTAP	733	1048	5.3%	BBT	J. Am. Chem. Soc. 2018, 140, 1715-1724.
IR-FP8P	748	1040	0.6%	BBT	Chem.Mater. 2020, 32, 2061-2069.
CQ-4T	750	~ 1050	0.1%	BBT	Adv. Funct. Mater. 2020, 30, 1906343.
Q-FlTBBTTFl	814	~ 1090	< 0.1%	BBT	ACS Appl. Mater. Interfaces 2016, 8, 15937−15947.
IR-FEP	780	~ 1050	2.0%	BBT	Adv. Mater. 2017, 29, 1605497.
IR-E1	830	1071	0.7%	BBT	Adv. Mater. 2017, 29, 1605497.
IR-FGP	745	1050	1.9%	BBT	Proc. Natl Acad. Sci. USA. 2017, 114, 962-967.
SWIR-WAZABY-01	738	720-1200	2.54% in 10% mouse plasma	BBT	Bioconjugate Chem. 2020, 31, 1088-1092.
TPA-T-TQ	~ 780	~ 1020	-----	PTQ	ACS Nano. 2017, 11,

NPs					7177-7188.
TQ-BPN dots	630	810	>900 nm, 2.8%	PTQ	ACS Nano. 2018, 12, 7936-7945.
DPTQ-PTZ NPs	639	928	0.16%	PTQ	ACS Appl. Mater. Interfaces. 2020, 12, 20281-20286.

Supplementary Table 3. Details of imaging parameters used in each fluorescent image of this study.

Figures	Dyes	Dose	Laser (nm/mWcm^{-2})	Filter sets	Exposure time(ms)
**Fig. 2h, 2i**	TPA-TQT/ CH-4T	-----	808/150	1100	------
**Fig. 3c**	FT-BBT/ FT-TQT	OD 0.1	808/180	1100<br>1250<br>1350	100<br>500<br>2000
**Supplementary Fig. 4**	FT-BBT/ FT-TQT/ ICG	-----	808/150	1100	200
**Fig. 3h, Supplementary Fig. 9**	FT-BBT<br>FT-TQT	1 mM	808/115	1300<br>1500	300, 500, 800<br>2000,3000, 4000
**Fig. 4d**	FT-TQT	50 µg	808/150	1000	3000
**Fig. 4f**	FT-TQT	200 µg	808/150	1000<br>1100<br>1200<br>1300<br>1400	100<br>200<br>300<br>30000<br>200000
**Fig. 4h, Supplementary Fig 12**	FT-TQT	200 µg	808/150	1400	200000
**Fig. 5b**	FT-TQT @FBS	200 µg	808/280	1400	10000
**Fig. 5d**	FT-TQT @FBS	100 µg	808/280	1100<br>1350	1000<br>3000
**Fig. 5f**	FT-TQT @FBS	200 µg	808/280	1100	500
**Fig. 6b, 6c**	FT-TQT @FBS	200 µg	808/280	1350	3000
**Supplementary Fig. 7**	FT-TQT	10 µM	808/115	1000	10

**Fig. 4a, Supplementary Fig. 10, 11**	FT-BBT<br>FT-TQT	200 μg	808/235	1250<br>1300<br>1400	2000<br>3000<br>10000
**Supplementary Fig. 15, 16**	FT-TQT<br>FT-TQT@FBS	100 μg<br>100 μg	808/90<br>808/90	1000<br>1000	30<br>30
**Supplementary Fig. 22e**	FT-TQT/ TPA-TQT	8.5 μM	808/100	1000	100
**Supplementary Fig. 22f**	TPA-TQT<br>FT-TQT	------	808/100	1000	50<br>20

Synthesis of TPA-TQT-TMS.

Zinc dust (369.4 mg, 5.65 mmol) and NH_4Cl (90 mg, 1.68 mmol) were added to a stirred solution of TPA-BT-NO_2 (66 mg, 0.05 mmol) in dichloromethane (10 mL) and 90% methanol (5 mL) under nitrogen atmosphere. After being stirred at room temperature for 5 h, the solution was filtered through Celite pad, diluted with dichloromethane (20 mL), and washed with water, saturated aqueous $NaHCO_3$, and saturated aqueous brine (10 mL). The organic phase was dried over anhydrous Na_2SO_4, filtered and concentrated under vacuum to afford a yellow solid which was utilized for the next step without further purification. To a yellow solution in anhydrous acetic acid (5 mL) was added 1,2-di(thiophen-2-yl) ethane-1,2-dione (16 mg, 0.07 mmol). The mixture was heated in an oil bath at 110°C for overnight. The reaction mixture was allowed to cool down to room temperature, extracted with dichloromethane. The combined organic layer was washed with water and concentrated *in vacuo*. The residue was purified by chromatography (petroleum ether: ethyl acetate, 5:1) to yield the product TPA-TQT-TMS as a dark green solid (20 mg, two step 20% yield). MALDI-TOF-MS was used to identify the product. MALDI-TOF-MS Calcd for: [$C_{84}H_{98}N_6O_8S_3Si_4$] (M.W.): 1526.569. Found: 1526.534. ^{1}H NMR (400 MHz, $CDCl_3$) δ 7.93 (d, J = 8.5 Hz, 4H), 7.55 (d, J = 4.2 Hz, 2H), 7.50 (d, J = 3.4 Hz, 2H), 7.28-7.26 (m, 5H), 7.20-7.13 (m, 15H), 7.05 – 7.01 (m, 2H), 4.20 (t, J = 8.0 Hz, 8H), 2.95 (t, J = 8.0 Hz, 8H), 2.64 (t, J = 8.0 Hz, 8H), 1.00 (t, J = 8.0 Hz, 8H), 0.05 (s, 36H). ^{13}C NMR (126 MHz, $CDCl_3$) δ 172.69, 152.79, 147.63, 145.46, 145.28, 142.34, 135.16, 133.55, 130.08, 129.93, 127.46, 127.36, 127.07, 124.66, 120.91, 62.24, 35.70, 29.99, 16.89, -1.90.

Synthesis of TPA-TQT.

To a solution of compound TPA-TQT-TMS (10 mg, 0.006 mmol) in DCM (3 mL) was cooled at 0°C. Trifluoroacetic acid (3 mL) was added and the reaction mixture was stirred at 0°C for 2 h. The reaction mixture was slowly warmed to ambient temperature. The solvent was removed in vacuo and the crude product was washed by dichloromethane to yield the desired compound as a green solid which was used for the next step without further purification.

To a green solution in 1 mL dry DMF was added TBTU (26 mg 0.081 mmol), DIPEA (32 mg, 0.25 mmol). Stirred for 30 min and then added taurine (31 mg, 0.25 mmol). The reaction solution was stirred over night at room temperature under a nitrogen atmosphere. After the reaction finished, 1 mL of water was added and stirred for 1 h to quench the excess TBTU. Finally, SHMADZU Summit high-performance liquid chromatography (HPLC) system was performed on a LC-20AR Instrument with PDA detection (column: XBridge Protein BEH C4, 5 μm, 250 mm × 10 mm; mobile phase: water/acetonitrile with 0.1 % TFA), 3 mL/min flow rate, 254 nm and 690 nm detection wavelength were used to purify the reaction. 10 mg TPA-TQT (Yield 72%) was got as a green sold. MALDI-TOF-MS was used to identify the product. MALDI-TOF-MS Calcd for: [$C_{72}H_{70}N_{10}O_{16}S_7$] (M.W.): 1555.826. Found: 1555.317. ^{1}H NMR (400 MHz, DMSO-d_6) δ 7.90 (d, J = 4.9 Hz, 2H), 7.87 – 7.83 (m, 6H), 7.41 (d, J = 3.7 Hz, 2H), 7.22 (d, J = 8.3 Hz, 8H), 7.15 – 7.11 (m, 2H), 7.08 (d, J = 8.2 Hz, 10H), 3.33 (m, 8H), 2.81 (t, J = 7.8 Hz, 8H), 2.56 (t, J = 7.7 Hz, 8H), 2.38 (t, J = 7.7 Hz, 8H). ^{13}C NMR (126 MHz, DMSO-d_6) δ 171.45, 153.03, 148.01, 146.29, 145.32, 142.15, 137.12,

135.36, 134.50, 132.04, 130.98, 129.95, 128.38, 127.97, 127.70, 125.20, 120.58, 51.13, 37.61, 36.01, 31.03.

Synthesis of FT-BT-NO_2.

A 50 mL schlenk flask was charged with compound 4,7-bis(5-bromothiophen-2-yl)-5,6-dinitrobenzo[c][1,2,5]thiadiazole (200 mg, 0.3 mmol), bis(2-(trimethylsilyl)ethyl) 6,6'-(2-(4,4,5,5-tetramethyl-1,3,2-dioxaborolan-2-yl)-9H-fluorene-9,9-diyl)dihexanoate (430 mg, 0.6 mmol), and dichloro[1,1'-bis(diphenyl phosphino) ferrocene]-palladium dichloromethane adduct (42 mg, 0.006 mmol), aqueous K_2CO_3 (102 mg, 0.74 mmol, 1 mL) in THF (6 mL) under an inert atmosphere (N_2). The resulting mixture was further degassed with an N_2 stream for 10 min and heated in an oil bath at 80°C for overnight. The reaction was allowed to cool to room temperature and extracted with EtOAc (2 × 20 mL). The combined organic layers were washed with water (50 mL) and sat. brine (100 mL). After drying over anhydrous Na_2SO_4 and removal of the solvents under reduced pressure, the residue was purified by column chromatography on silica gel (the residue was purified by column chromatography on silica gel (petroleum ether: ethyl acetate = 4:1 v/v) to yield the product as a purple solid (170 mg, 36% yield).) to yield the product as a purple solid (170 mg, 36% yield). ^{1}H NMR (400 MHz, Chloroform-d) δ 7.78-7.70 (m, 6H), 7.65-7.62 (m, 2H), 7.58-7.56 (m, 2H), 7.53-7.51 (m, 2H), 7.42-7.34 (m, 6H), 4.17 – 4.07 (t, J = 7.5 Hz, 8H), 2.12 (t, J = 7.5 Hz, 8H), 2.09 – 2.00 (m, 8H), 1.41 (p, J = 7.6 Hz, 8H), 1.13 (p, J = 7.5 Hz, 8H), 0.98 – 0.90 (m, 8H), 0.67 (tq, J = 13.8, 7.9 Hz, 8H), 0.03 (s, 36H). ^{13}C NMR (101 MHz, $CDCl_3$) δ 173.93, 152.09, 151.81, 151.36, 150.60, 142.09, 141.45, 140.31, 132.17,

131.97, 128.40, 127.72, 127.12, 125.40, 123.99, 122.87, 120.64, 120.37, 120.24, 120.06, 62.37, 55.18, 40.22, 34.40, 29.47, 24.63, 23.46, 17.28, -1.47.

Synthesis of FT-BBT-TMS.

Zinc dust (500 mg, 7.6 mmol) and NH_4Cl (122 mg, 2.28 mmol) were added to a stirred solution of FT-BT-NO_2 (100 mg, 0.06 mmol) in dichloromethane (8 mL) and 90% methanol (10 mL) under nitrogen atmosphere. After being stirred at room temperature for 4 h, the solution was filtered through Celite pad, diluted with dichloromethane (30 mL), and washed with water (10 mL × 3), saturated aqueous $NaHCO_3$, and saturated aqueous brine (10 mL). The organic phase was dried over anhydrous Na_2SO_4, filtered and concentrated under vacuum to afford a yellow solid (Y1) which was used for the next step without further purification.

N-thionylaniline (0.08 mL, 0.72 mmol) and chlorotrimethylsilane (0.06 mL, 0.72 mmol) were added to a dark yellow solution of Y1 (110 mg, 0.07 mmol) in anhydrous pyridine (2 mL) under nitrogen atmosphere. The mixture was heated in an oil bath at 80°C for overnight. The reaction mixture was allowed to cool down to room temperature, poured into iced water (20 mL), extracted with dichloromethane. The combined organic layer was washed with water (3 × 15 mL), saturated aqueous brine (30 mL), dried over anhydrous $MgSO_4$ and concentrated in vacuo. The residue was purified by column chromatography on silica gel (petroleum ether: ethyl acetate = 3:1 v/v) to yield the product as a green solid (40 mg, 36% yield). MALDI-TOF-MS Calcd for: [$C_{84}H_{110}N_4O_8S_4Si_4$] (M.W.): 1542.628. Found: 1542.586. ^{1}H NMR (400 MHz, Chloroform-*d*) δ 9.06-9.02 (m, 2H), 7.85-7.81 (m, 2H), 7.79 – 7.74 (m, 6H), 7.62 (m,

2H), 7.42-7.34 (m, 6H), 4.15 – 4.05 (m, 8H), 2.12 (q, *J* = 5.8, 4.1 Hz, 16H), 1.45 (p, *J* = 7.5 Hz, 8H), 1.18 (p, *J* = 7.4 Hz, 8H), 0.99 – 0.90 (m, 8H), 0.85-0.65 (m, 8H), 0.01 (s, 36H). ^{13}C NMR (101 MHz, $CDCl_3$) δ 173.92, 151.26, 151.23, 150.62, 149.91, 141.48, 140.62, 137.34, 134.13, 133.11, 127.49, 127.08, 125.11, 124.35, 122.87, 120.30, 119.95, 119.93, 113.24, 62.34, 55.19, 40.24, 34.47, 29.53, 24.66, 23.52, 17.27, -1.49.

Synthesis of compound FT-TQT-TMS.

2,2′-Thenil (22 mg, 0.1 mmol) was added to a dark yellow solution of compound Y1 (100 mg, 0.066 mmol) in acetic acid (5 mL) under nitrogen atmosphere. The mixture was heated in an oil bath at 100°C for overnight. The reaction mixture was allowed to cool down to room temperature, poured into iced water (20 mL), extracted with ethyl acetate. The combined organic layer was washed with water (3 × 15 mL), saturated aqueous brine (30 mL), dried over anhydrous Na_2SO_4 and concentrated in vacuo. The residue was purified by column chromatography on silica gel (petroleum ether: ethyl acetate = 3:1 v/v) to yield the product as a green solid (35 mg, 31% yield). ^{1}H NMR (400 MHz, Chloroform-*d*) δ 9.06 (d, *J* = 4.1 Hz, 2H), 7.88 – 7.85 (m, 4H), 7.81 – 7.75 (m, 4H), 7.72 – 7.71 (m, 2H), 7.67 – 7.62 (m, 4H), 7.42 – 7.36 (m, 6H), 7.17 -7.14 (m, 2H), 4.11 (t, *J* = 8.0 Hz, 8H), 2.14 – 2.09 (m, 16H), 1.44 (p, *J* = 7.4 Hz, 8H), 1.17 (p, *J* = 7.5 Hz, 8H), 0.93 (t, *J* = 8.0 Hz, 8H), 0.84 – 0.67 (m, 8H), 0.01 (m, 36H). ^{13}C NMR (126 MHz, $CDCl_3$) δ 173.39, 151.58, 150.68, 150.06, 149.72, 145.18, 141.26, 140.63, 140.30, 134.91, 134.29, 133.67, 133.18, 131.24, 130.30, 127.28, 126.89, 126.62, 124.42, 122.78, 122.41, 119.81, 119.75, 119.68, 119.42, 61.85, 54.60, 39.96, 33.95, 29.14, 24.19, 23.07, 16.82, -1.96.

Synthesis of FT-BBT.

To a solution of compound FT-BBT-TMS (5 mg, 0.004 mmol) in DCM (3 mL) was cooled at 0°C. Trifluoroacetic acid (1 mL) was added and the reaction mixture was stirred at 0°C for 6 h. The reaction mixture was slowly warmed to ambient temperature. The solvent was removed in vacuo and the crude product was washed by dichloromethane to yield the desired compound as a green solid which was used for the next step without further purification.

To a green solution in 1 mL dry DMF was added TBTU (13 mg, 0.04 mmol), DIPEA (16 mg, 0.12 mmol). Stirred for 30 min and then added taurine (16 mg, 0.13 mmol). The reaction solution was stirred over night at room temperature under a nitrogen atmosphere. After the reaction finished, 1 mL of water was added and stirred for 1 h to quench the excess TBTU. Finally, Shimadzu LC-20AR high-performance liquid chromatography (HPLC) system (Shimadzu Corporation, Japan), XBridge protein BEH C4, 10 mm × 250 mm preparative column, gradient elution starting from 30% acetonitrile and ending up with 90% acetonitrile (in water with 0.1%TFA) at 15 mins, 3 mL/min flow rate, 254 nm and 820 nm detection wavelength was used to purify the reaction. 4 mg FT-BBT (Yield 58%) was got as a green solid. ^{1}H NMR (400 MHz, DMSO-d_6) δ 9.05 (d, J = 3.9 Hz, 2H), 7.99 (brs, 2H), 7.95 – 7.89 (m, 4H), 7.88-7.81 (m, 4H), 7.64 (t, J = 5.5 Hz, 3H), 7.53 (s, 2H), 7.37 (dd, J = 5.5, 3.0 Hz, 3H), 3.23 (t, J = 6.8 Hz, 8H), 2.57-2.53 (m, 8H), 2.24 – 1.98 (m, 8H), 1.84 (t, J = 7.3 Hz, 8H), 1.25 (p, J = 7.2 Hz, 8H), 1.11-0.95 (m, 8H), 0.68 – 0.48 (m, 8H). ^{13}C NMR (126 MHz, DMSO) δ 172.09, 151.67, 150.89, 150.84, 149.11, 141.43, 140.49, 137.03, 134.19, 132.95,

128.09, 127.56, 125.43, 125.21, 123.46, 121.12, 120.60, 120.02, 112.63, 55.34, 51.08, 35.99, 35.85, 29.59, 25.48, 23.96. MALDI-TOF-MS Calcd for: [$C_{82}H_{88}N_8O_{10}S_9$]: 1570.361. Found: 1570.345.

Synthesis of FT-TQT.

The same reaction as described above to prepare FT-BBT was used, and FT-TQT was obtained as a yellow green solid (5 mg, 60%). ^{1}H NMR (400 MHz, DMSO-d_6) δ 9.05 (brs, 2H), 8.02 – 7.91 (m, 5H), 7.90-7.83 (d, *J* = 5.3 Hz, 5H), 7.65-7.56 (m, 5H), 7.56-7.49 (m, 2H), 7.43-7.35 (m, 4H), 7.29-7.23 (m, 2H), 3.28-3.19 (m, 8H), 2.61-2.52 (m, 8H), 2.18-2.05 (m, 8H), 1.84 (t, *J* = 7.0 Hz, 8H), 1.31-1.20 (m, 8H), 1.13-1.00 (m, 8H), 0.74-0.48 (m, 8H). ^{13}C NMR (126 MHz, DMSO) δ 171.53, 151.12, 150.32, 149.13, 145.67, 140.71, 140.62, 140.02, 134.60, 134.53, 133.35, 132.82, 132.13, 131.66, 128.26, 127.56, 127.13, 124.46, 123.77, 123.02, 120.74, 120.09, 119.55, 119.08, 54.71, 50.59, 35.47, 35.35, 29.12, 24.94, 23.48. MALDI-TOF-MS Calcd for: [$C_{82}H_{88}N_8O_{10}S_9$]: 1728.380. Found: 1729.368.

NMR and MS spectra.

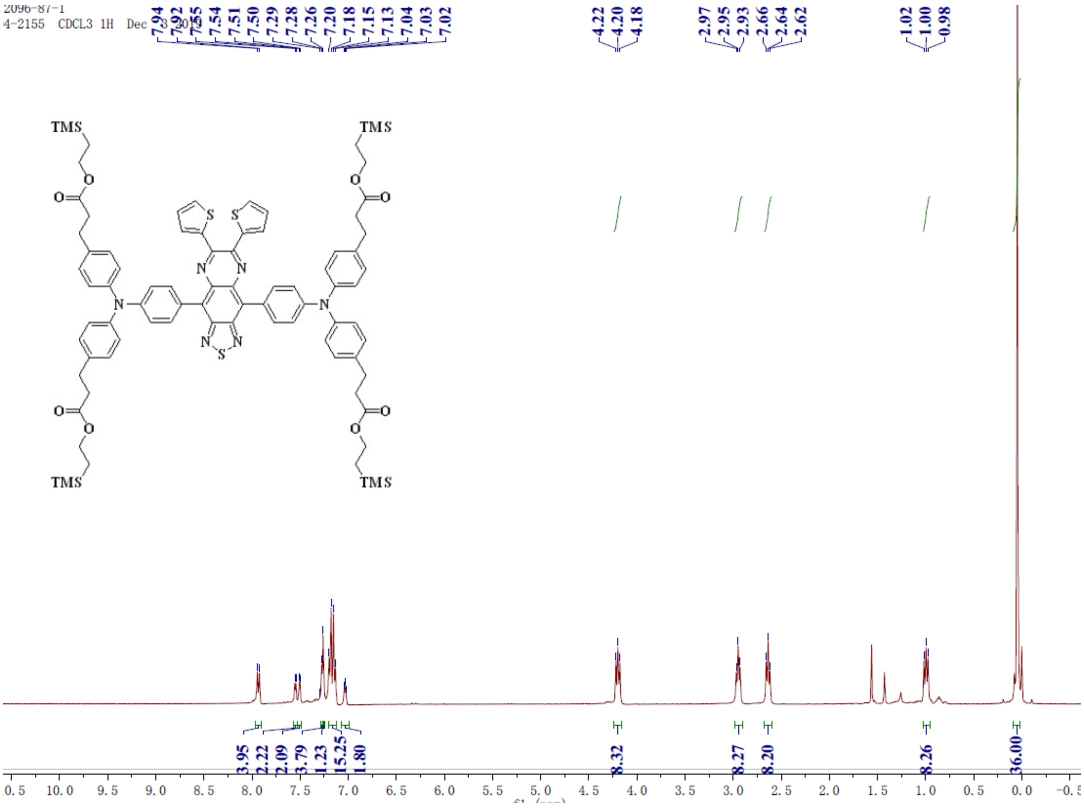

Supplementary Figure 28. The ^{1}H-NMR spectrum of TPA-TQT-TMS in *$CDCl_3$*.

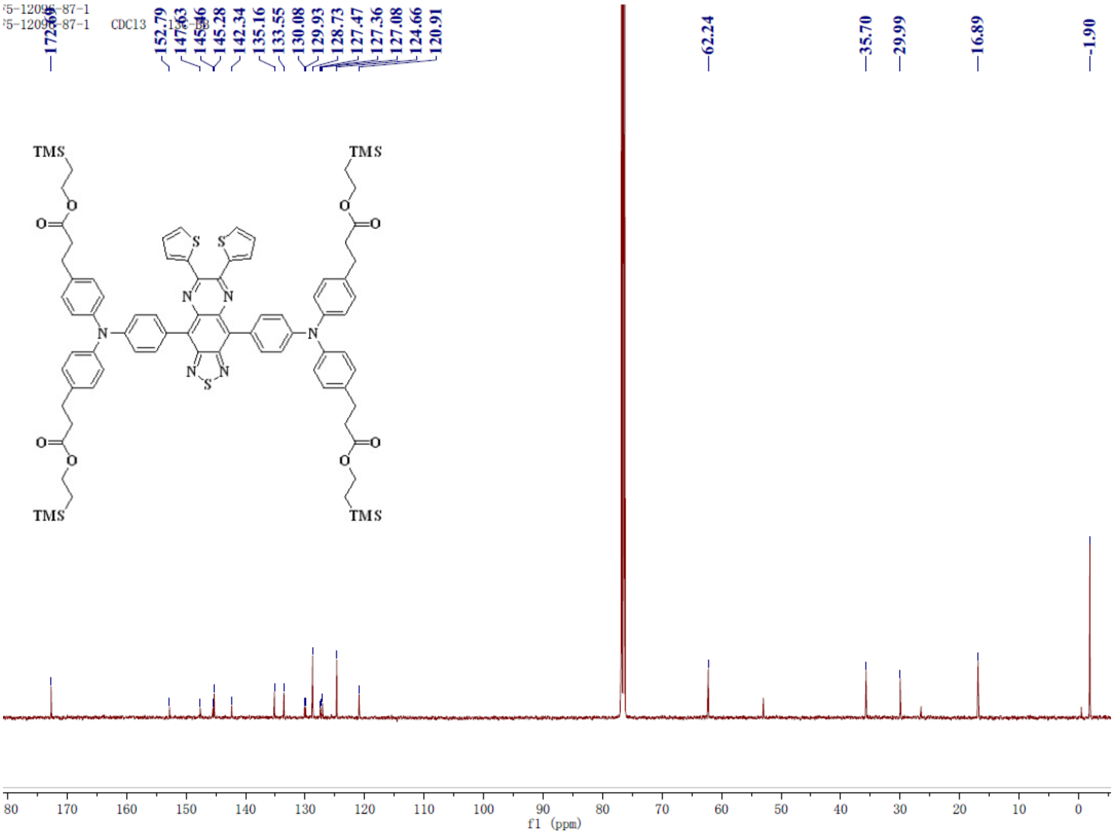

Supplementary Figure 29. The ^{13}C-NMR spectrum of TPA-TQT-TMS in *$CDCl_3$*.

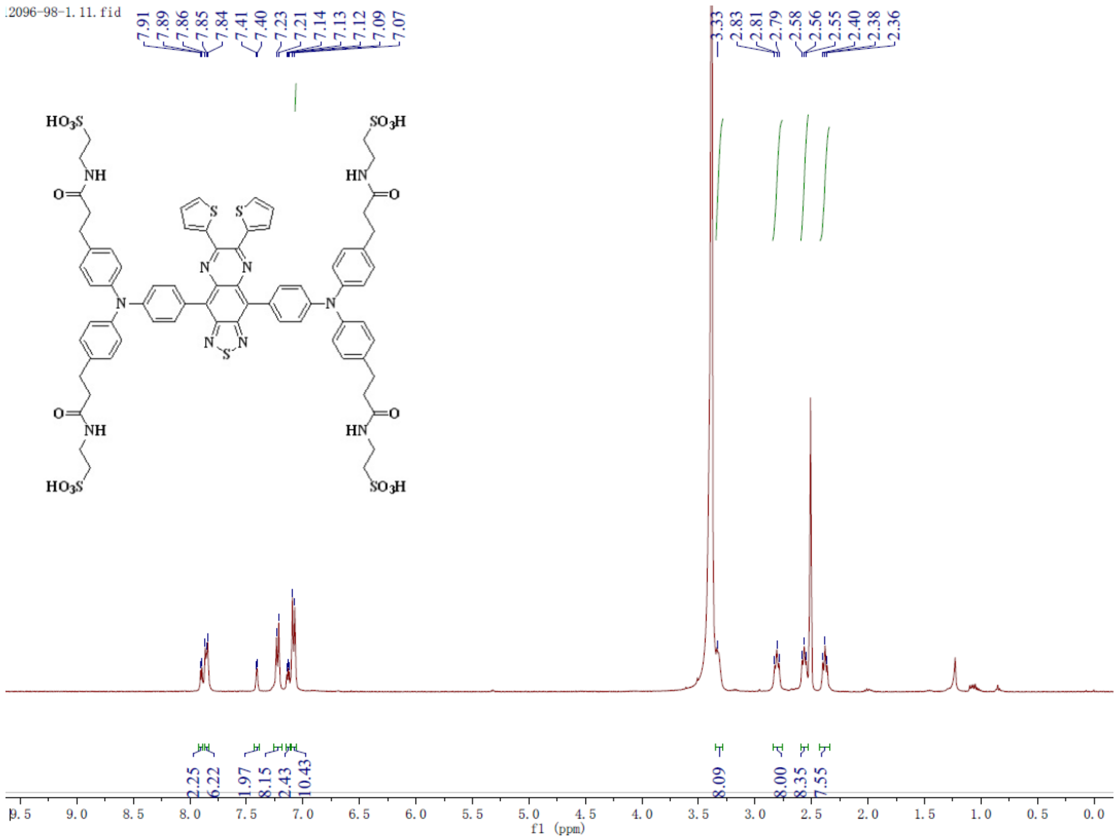

Supplementary Figure 30 The ^{1}H-NMR spectrum of TPA-TQT in *DMSO-d₆*.

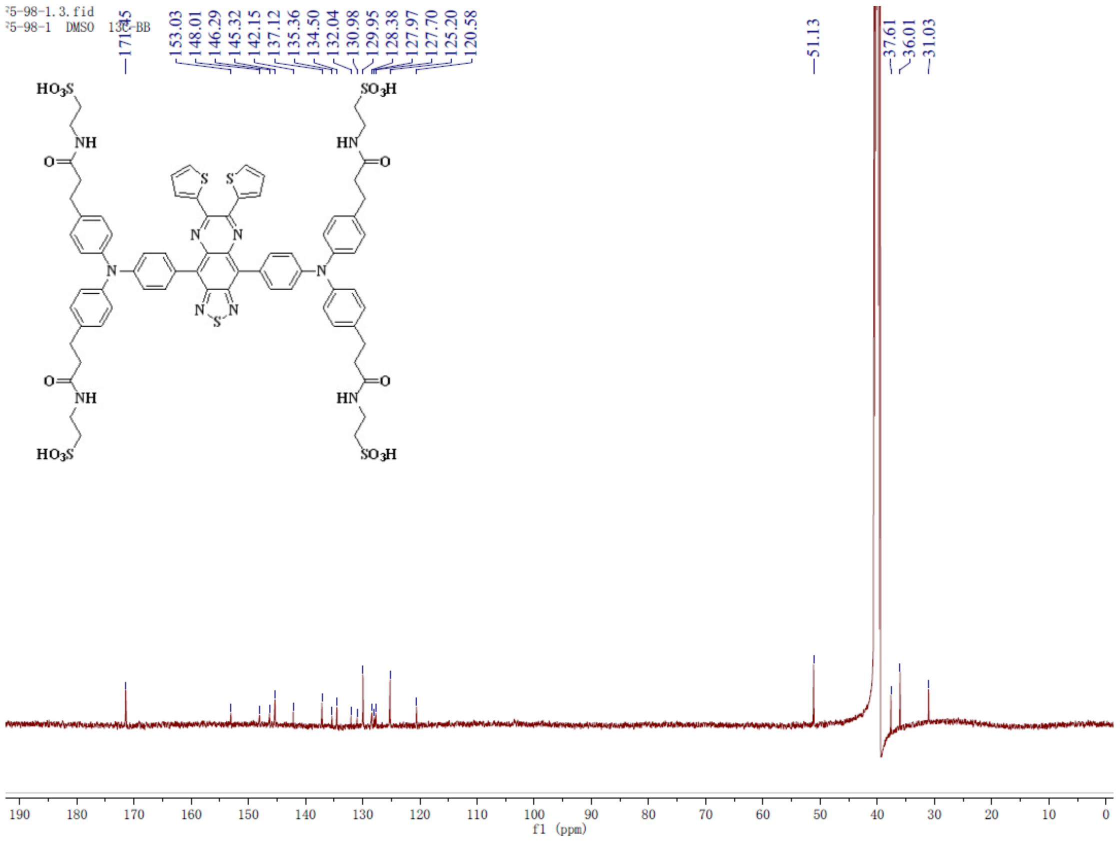

Supplementary Figure 31. The ^{13}C-NMR spectrum of TPA-TQT in *DMSO-d₆*.

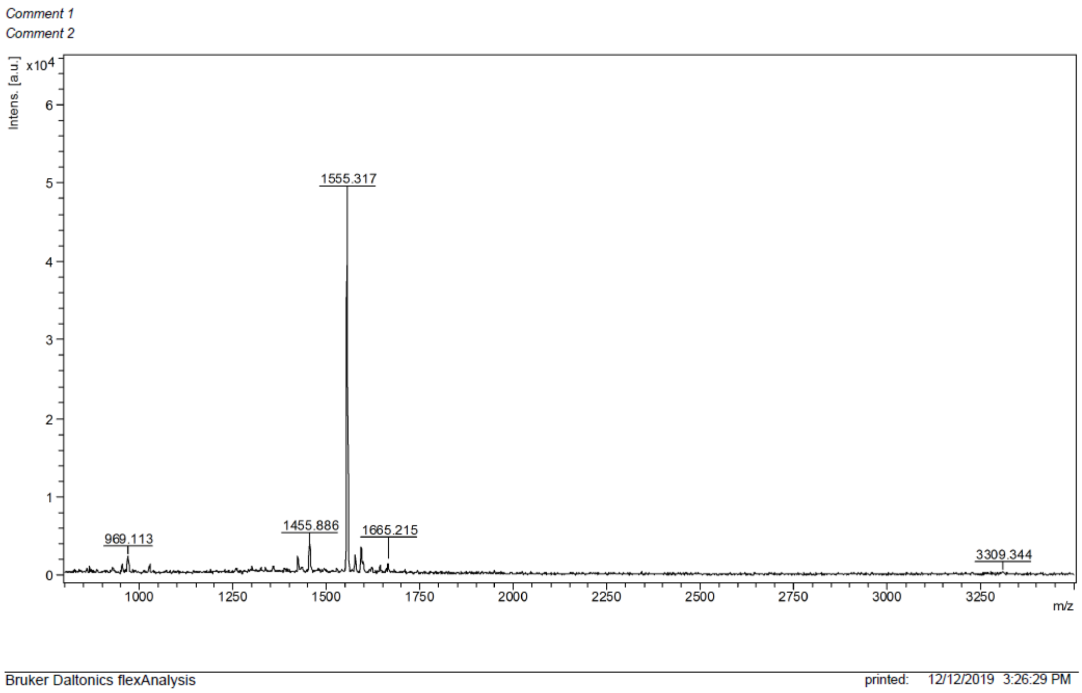

Supplementary Figure 32. The MALDI-TOF-MS of TPA-TQT.

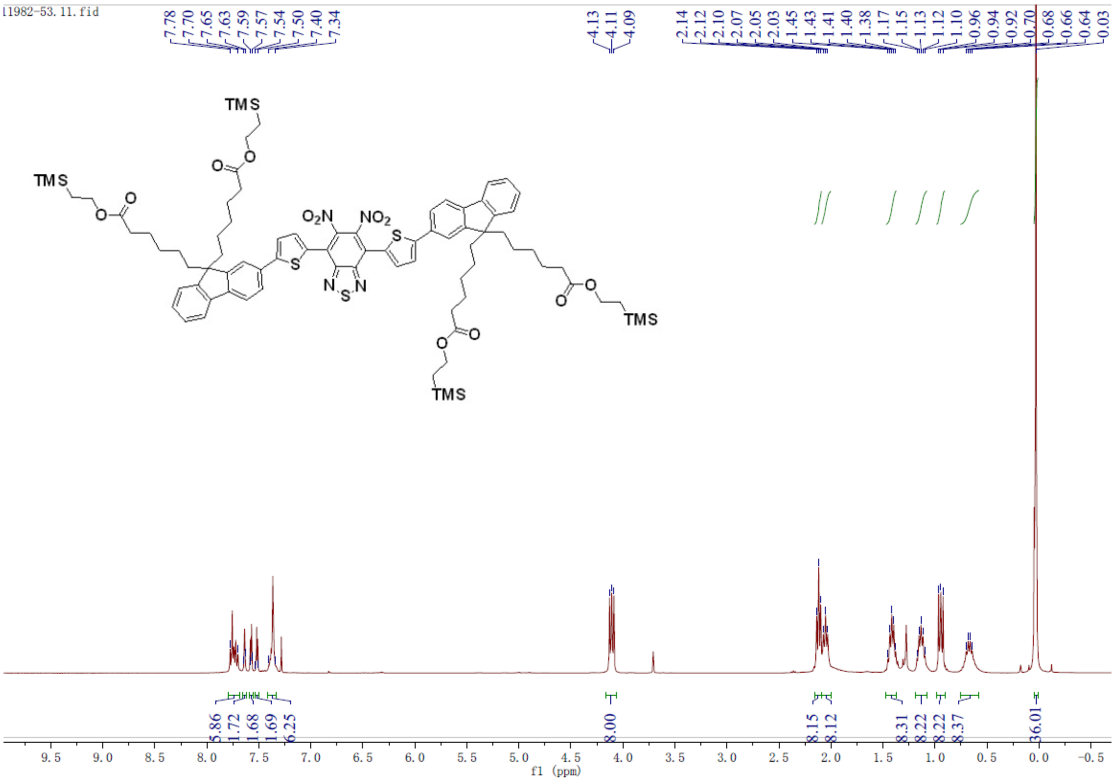

Supplementary Figure 33. The ^{1}H-NMR spectrum of FT-BT-NO_2 in *$CDCl_3$*.

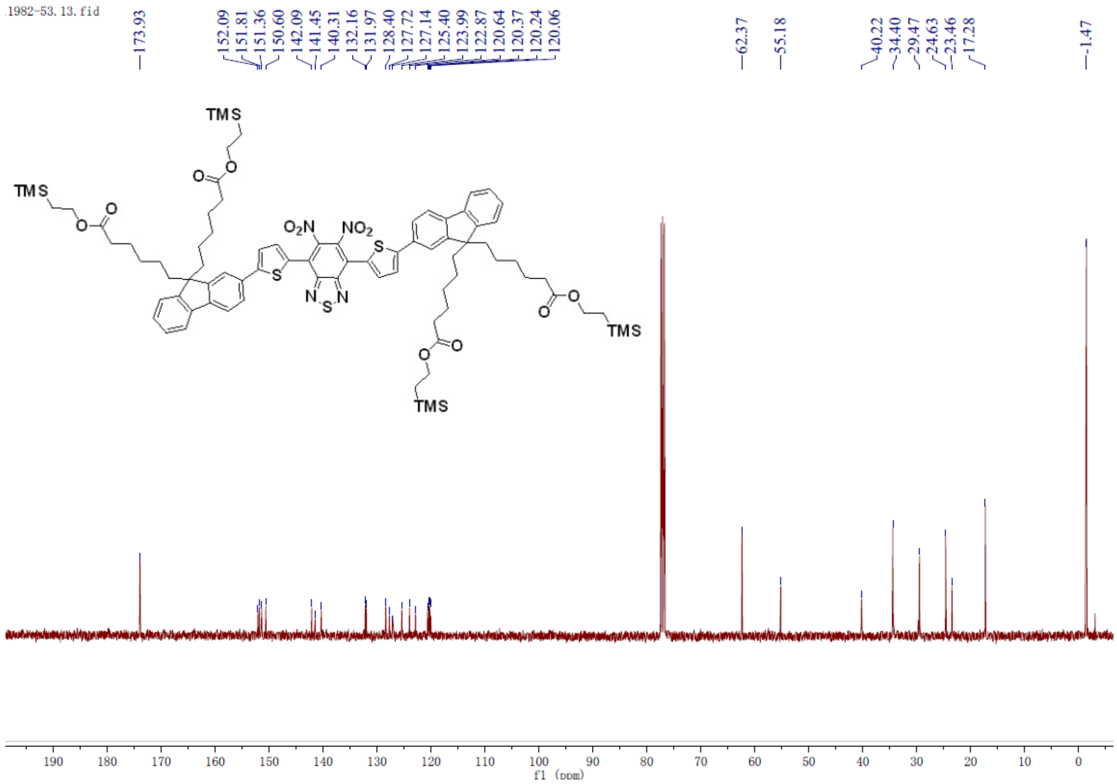

Supplementary Figure 34. The ^{13}C-NMR spectrum of FT-BT-NO_2 in *$CDCl_3$*.

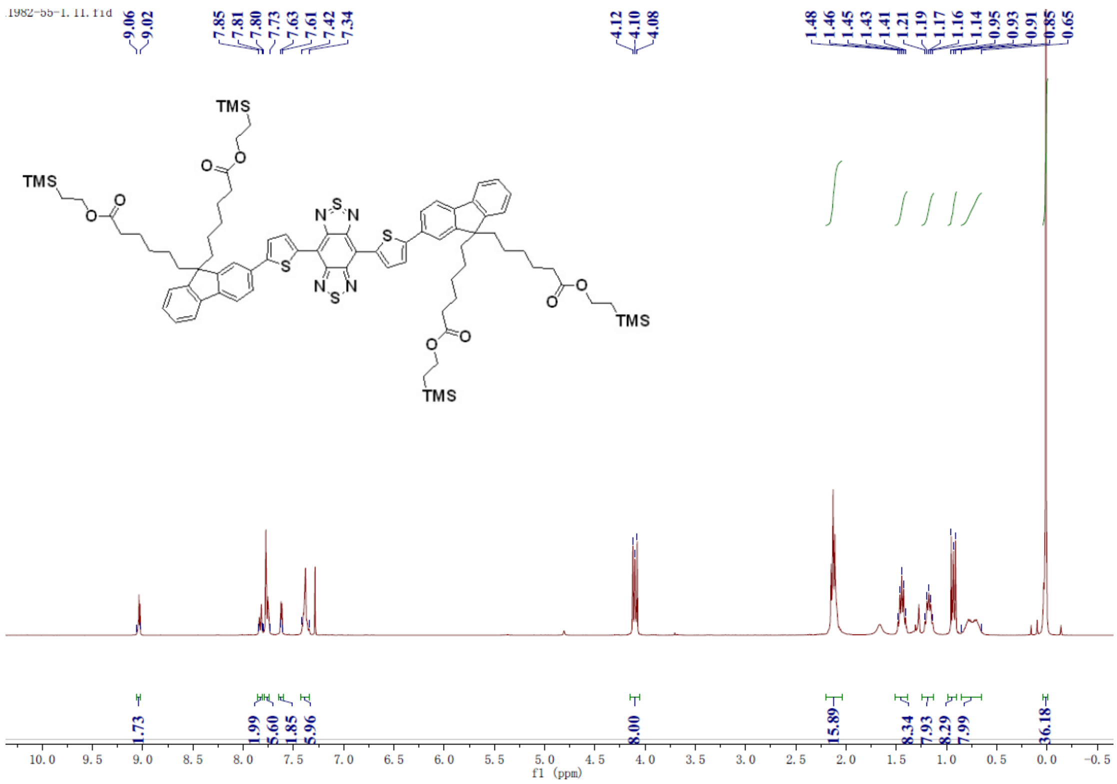

Supplementary Figure 35. The ^{1}H-NMR spectrum of FT-BBT-TMS in *$CDCl_3$*.

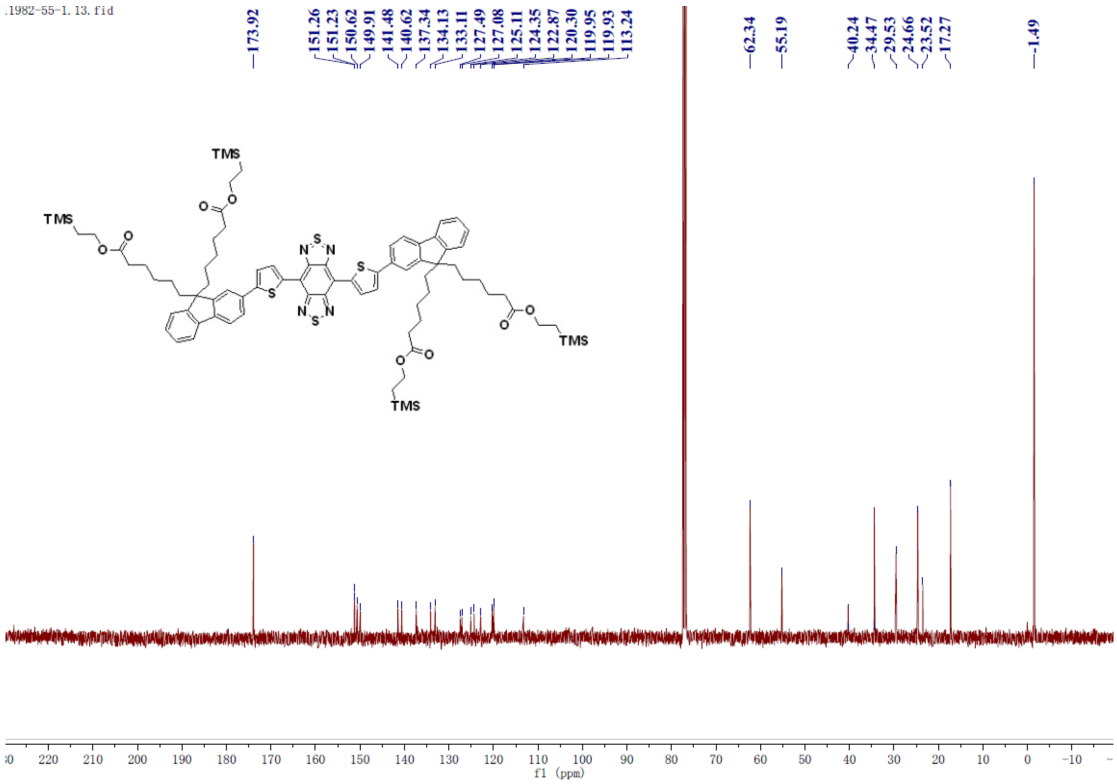

Supplementary Figure 36. The ^{13}C-NMR spectrum of FT-BBT-TMS in *$CDCl_3$*.

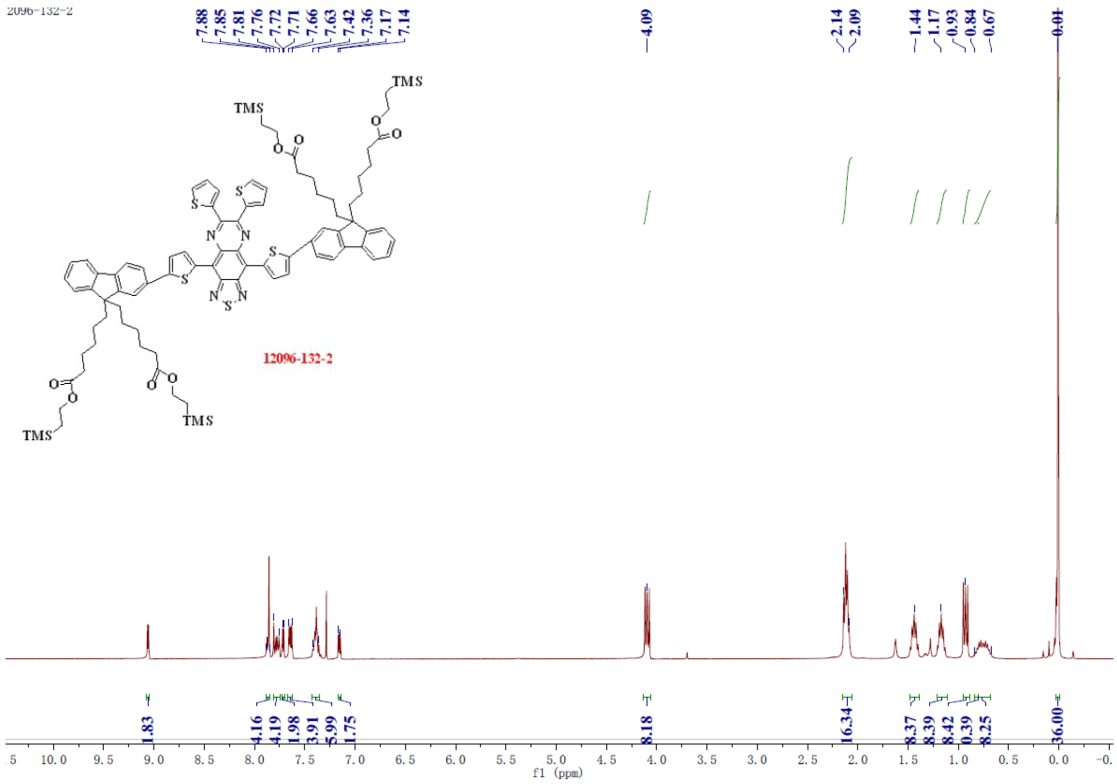

Supplementary Figure 37. The ^{1}H-NMR spectrum of FT-TQT-TMS in *$CDCl_3$*.

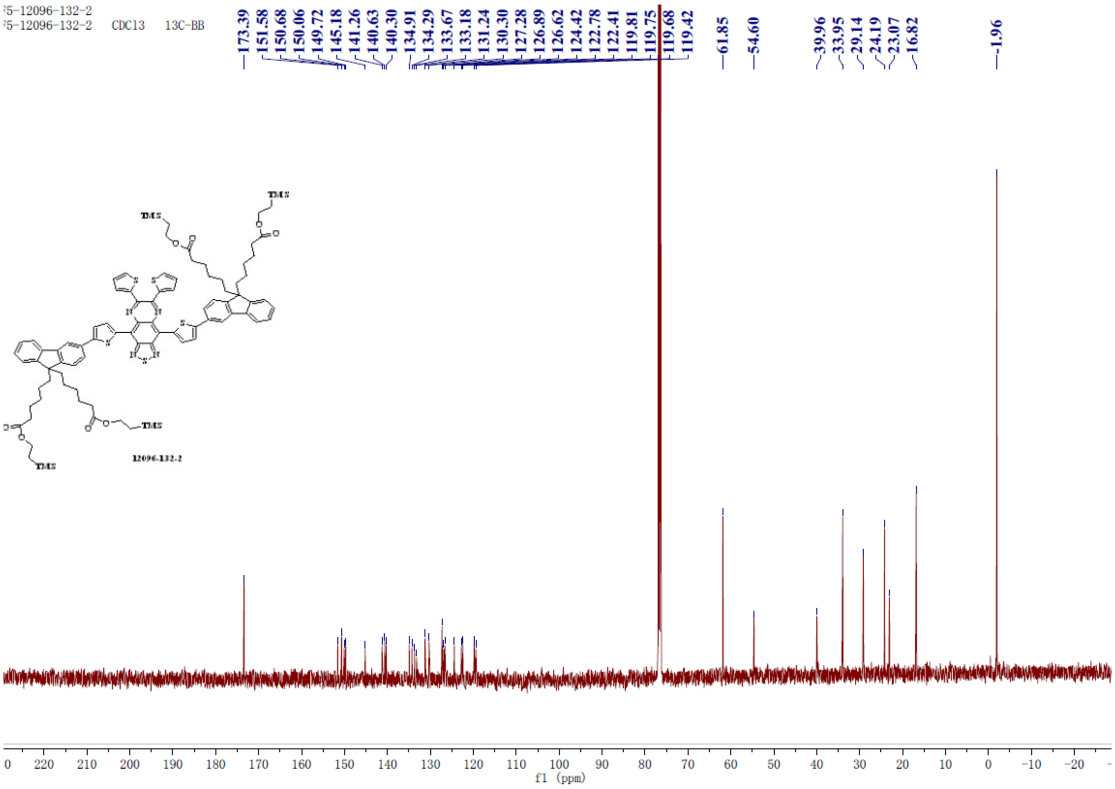

Supplementary Figure 38. The ^{13}C-NMR spectrum of FT-TQT-TMS in *$CDCl_3$*.

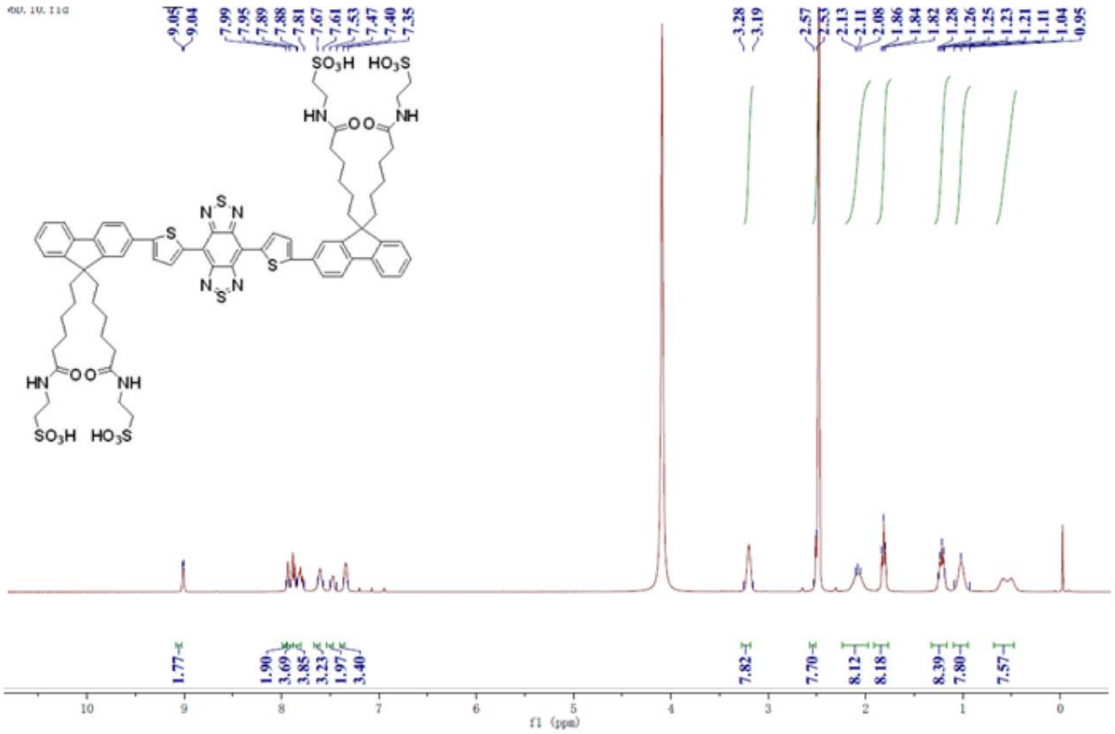

Supplementary Figure 39. The ^{1}H-NMR spectrum of FT-BBT in *DMSO-d_6*.

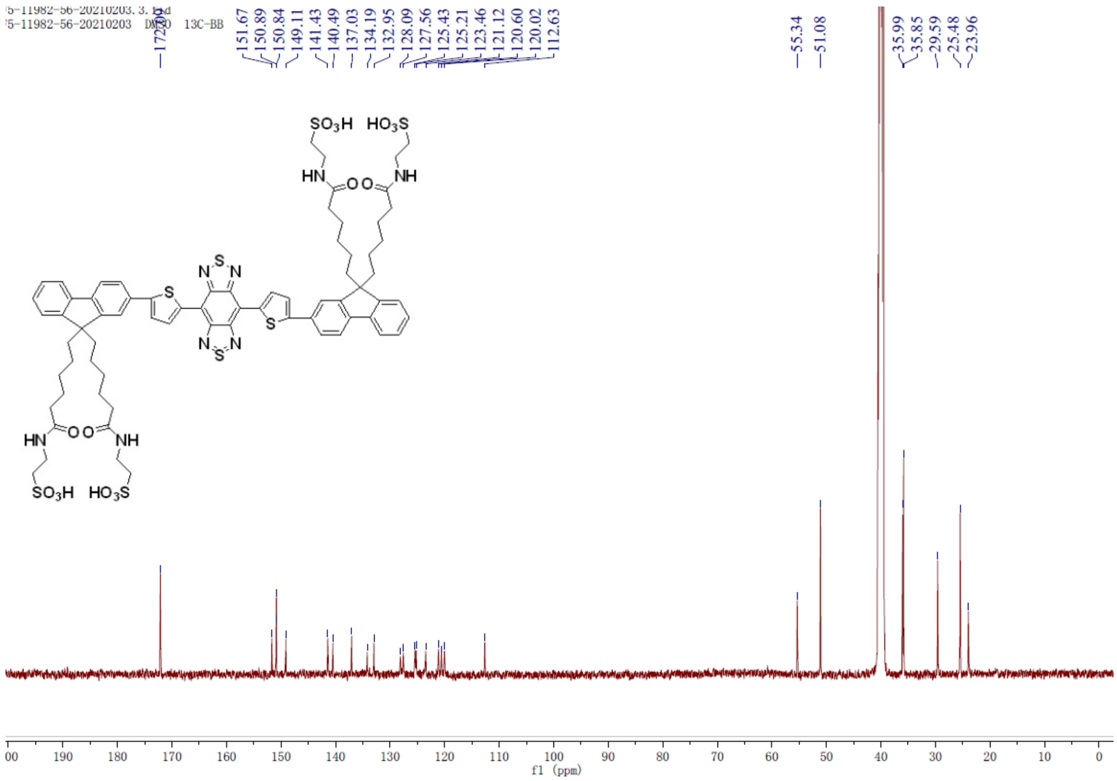

Supplementary Figure 40. The ^{13}C-NMR spectrum of FT-BBT in *DMSO-d$_6$*.

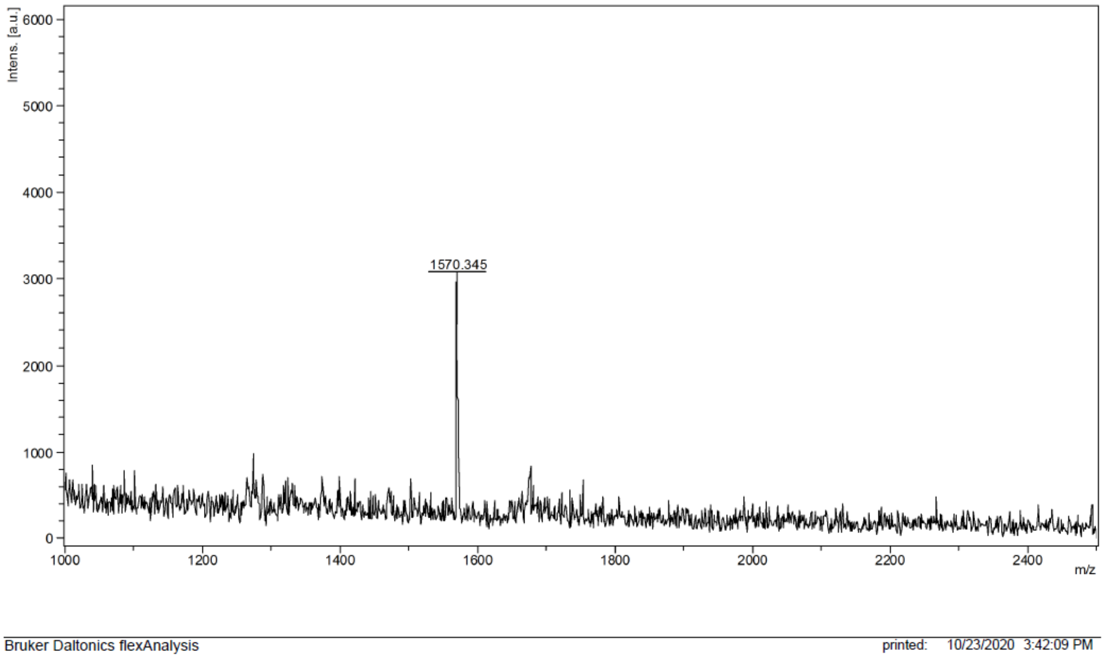

Supplementary Figure 41. The MALDI-TOF-MS of FT-BBT.

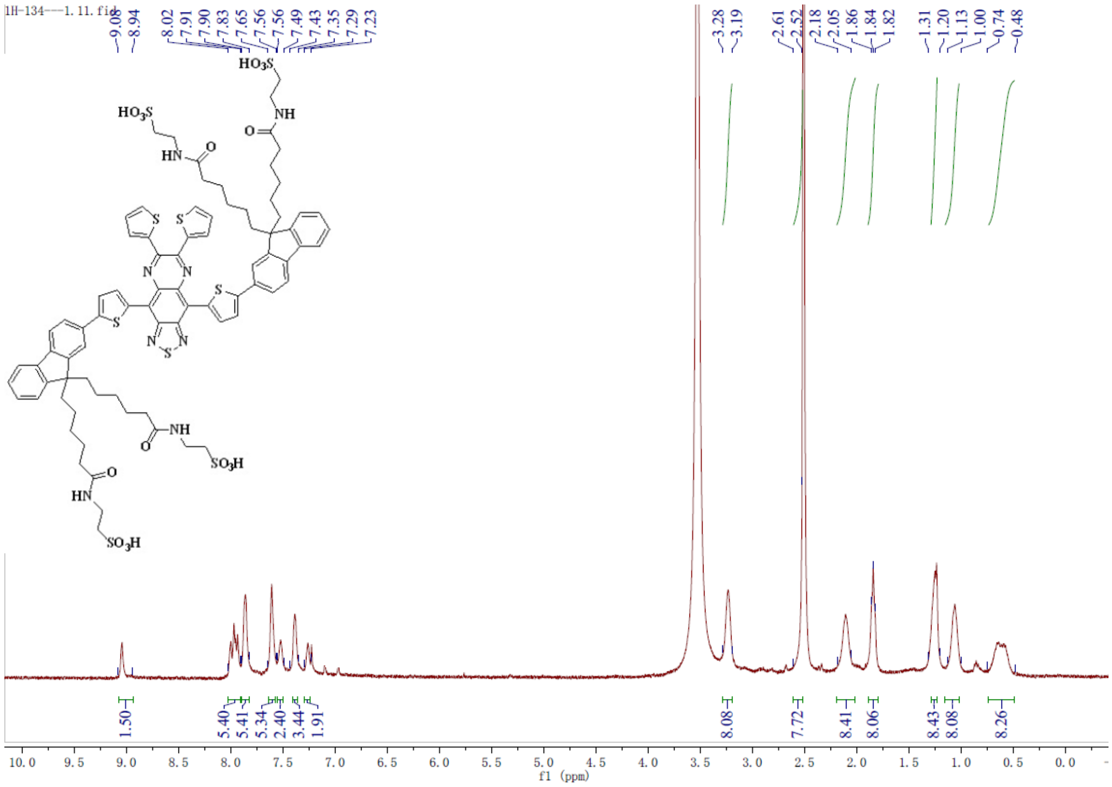

Supplementary Figure 42. The ^{1}H-NMR spectrum of FT-TQT in *DMSO-d_6*.

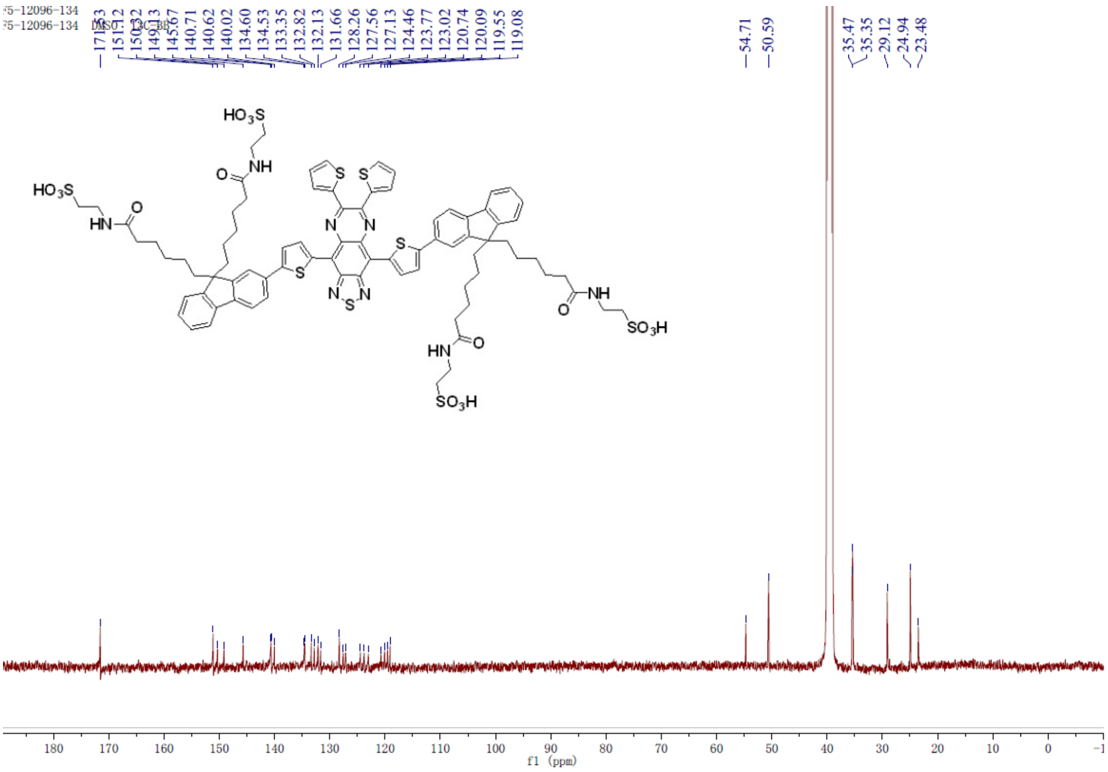

Supplementary Figure 43. The ^{13}C-NMR spectrum of FT-TQT in *DMSO-d_6*

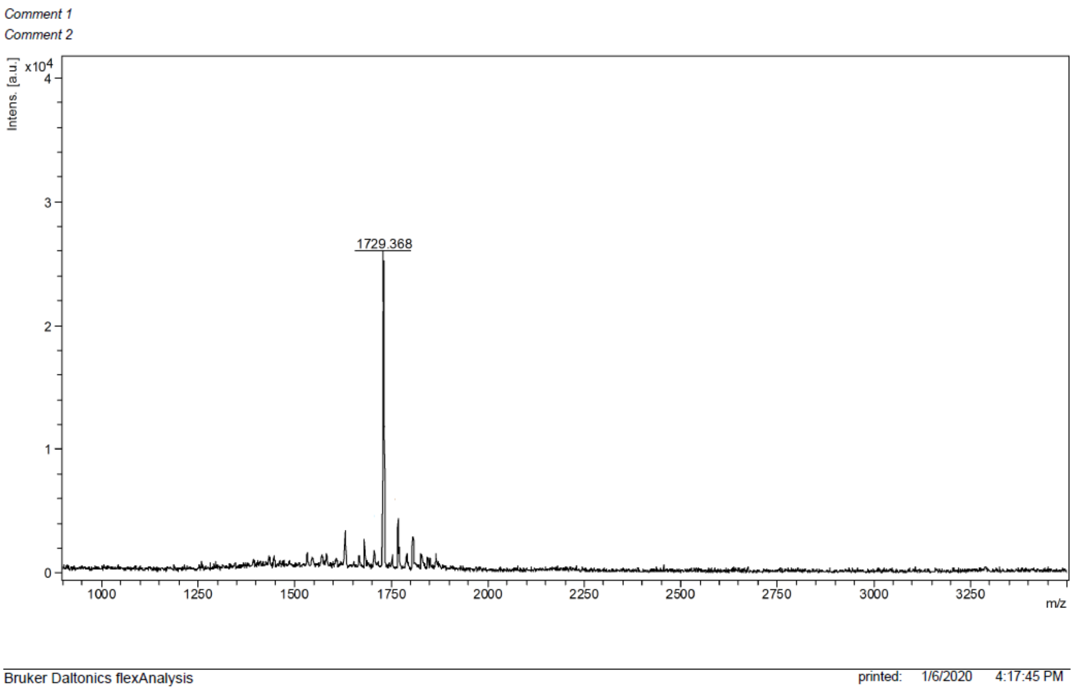

Supplementary Figure 44. The MALDI-TOF-MS of FT-TQT.

Supplementary References

1. Frangi, A. F., Niessen, W. J., Vincken, K. L., & Viergever, M. A. Multiscale vessel enhancement filtering. International conference on medical image computing and computer-assisted intervention. (Springer, Berlin, Heidelberg, 1998.)
2. Yang, Z. *et al*. Multi-parametric quantitative microvascular imaging with optical-resolution photoacoustic microscopy in vivo. *Opt. Express* **22**, 1500-1511 (2014).
