# Supplementary material for: Acceptor engineering for NIR-II dyes with high photochemical and biomedical performance
Source: Nat Commun. 2022 Jul 2;13:3815. doi: 10.1038/s41467-022-31521-y (PMC9250501; doi:10.1038/s41467-022-31521-y)
Supplement: Supplementary file 3 — Description of Additional Supplementary Files [file 41467_2022_31521_MOESM3_ESM.docx]

**Description of Additional Supplementary Files**

**Supplementary Movie 1.** Video of higher magnification NIR-II dynamic imaging was done to glean the details of tumor vasculatures in nude mice with xenograft osteosarcoma 143B (500 ms, 1100 LP) after FT-TQT@FBS complexes injection at a dose of 10 mg/kg.

**Supplementary Movie 2.** After 30 min administration of CA4P, dynamic tumor vasculatures NIR-II imaging of nude mice with xenograft osteosarcoma 143B was obtained after ～5 min FT-TQT@FBS p.i. (10 mg/kg, 3000 ms, 1350 LP).
