# Supplementary material for: Acceptor engineering for NIR-II dyes with high photochemical and biomedical performance
Source: Nat Commun. 2022 Jul 2;13:3815. doi: 10.1038/s41467-022-31521-y (PMC9250501; doi:10.1038/s41467-022-31521-y)
Supplement: Supplementary file 6 — Reporting Summary [file 41467_2022_31521_MOESM6_ESM.pdf]

nature portfolio

Corresponding author(s): Zhen Cheng

Last updated by author(s): May 8, 2022

Reporting Summary

Nature Portfolio wishes to improve the reproducibility of the work that we publish. This form provides structure for consistency and transparency in reporting. For further information on Nature Portfolio policies, see our Editorial Policies and the Editorial Policy Checklist.

Statistics

For all statistical analyses, confirm that the following items are present in the figure legend, table legend, main text, or Methods section.

n/a	Confirmed	
☐	☒	The exact sample size (n) for each experimental group/condition, given as a discrete number and unit of measurement
☐	☒	A statement on whether measurements were taken from distinct samples or whether the same sample was measured repeatedly
☐	☒	The statistical test(s) used AND whether they are one- or two-sided<br>*Only common tests should be described solely by name; describe more complex techniques in the Methods section.*
☒	☐	A description of all covariates tested
☐	☒	A description of any assumptions or corrections, such as tests of normality and adjustment for multiple comparisons
☐	☒	A full description of the statistical parameters including central tendency (e.g. means) or other basic estimates (e.g. regression coefficient) AND variation (e.g. standard deviation) or associated estimates of uncertainty (e.g. confidence intervals)
☐	☒	For null hypothesis testing, the test statistic (e.g. F, t, r) with confidence intervals, effect sizes, degrees of freedom and P value noted<br>*Give P values as exact values whenever suitable.*
☒	☐	For Bayesian analysis, information on the choice of priors and Markov chain Monte Carlo settings
☒	☐	For hierarchical and complex designs, identification of the appropriate level for tests and full reporting of outcomes
☒	☐	Estimates of effect sizes (e.g. Cohen's d, Pearson's r), indicating how they were calculated

Our web collection on statistics for biologists contains articles on many of the points above.

Software and code

Policy information about availability of computer code

Data collection	Lab Solutions (Version 1.0.7503. 20679); Shimadzu Common Container (Version 2.70); SoftMax Pro 7.1.lnk(Version 7.1.1); Lightfield (Version 6.11.4).
Data analysis	ChemBioDraw (Version 14.0); Origin (Version 9.6); Graphpad Prism (Version 8.0.2); Image J (Version1.8.0); MestReNova (Version 12.0.0); Adobe Illustrator CC 2018 (Version 22.1.0). Density function theory (DFT) calculations was performed using Gaussian 03 revision C.02 software andB3LYP method and 6-31G* basis set.

For manuscripts utilizing custom algorithms or software that are central to the research but not yet described in published literature, software must be made available to editors and reviewers. We strongly encourage code deposition in a community repository (e.g. GitHub). See the Nature Portfolio guidelines for submitting code & software for further information.

Data

Policy information about availability of data

All manuscripts must include a data availability statement. This statement should provide the following information, where applicable:

- Accession codes, unique identifiers, or web links for publicly available datasets
- A description of any restrictions on data availability
- For clinical datasets or third party data, please ensure that the statement adheres to our policy

The main data supporting the results in this study are available within the paper and its Supplementary Information. The authors could provide additional data related to this paper with reasonable request.

Field-specific reporting

Please select the one below that is the best fit for your research. If you are not sure, read the appropriate sections before making your selection.

☒ Life sciences ☐ Behavioural & social sciences ☐ Ecological, evolutionary & environmental sciences

For a reference copy of the document with all sections, see nature.com/documents/nr-reporting-summary-flat.pdf

Life sciences study design

All studies must disclose on these points even when the disclosure is negative.

Sample size	Animal sample size (n) was determined by the number of biological replicates necessary for ensuring statistical significance. The sample size (n) of each experiment are provided in the corresponding figure legends and methods in the paper and supplementary information. Sample sizes were chosen to support meaningful conclusions. Sample sizes were chosen based on similar published studies, with at least n=3 for biological independent samples/organisms. Ref: Nature communications, 11, 1-11 (2020)
Data exclusions	We excluded data from animals that died before experimental termination.
Replication	All of the experimental findings could be reliably reproduced. Most experiments were performed at least twice, with similar results. In animal experiments, at least three mice were included in each group.
Randomization	All samples/organisms were randomly allocated into experimental groups.
Blinding	No blinding experiments were designed in this experiment, since all data were collected and assembled by instrument software without interference. Investigators performed the same procedure to data process.

Reporting for specific materials, systems and methods

We require information from authors about some types of materials, experimental systems and methods used in many studies. Here, indicate whether each material, system or method listed is relevant to your study. If you are not sure if a list item applies to your research, read the appropriate section before selecting a response.

Materials & experimental systems

n/a	Involved in the study	
☒	☐	Antibodies
☐	☒	Eukaryotic cell lines
☒	☐	Palaeontology and archaeology
☐	☒	Animals and other organisms
☒	☐	Human research participants
☒	☐	Clinical data
☒	☐	Dual use research of concern

Methods

n/a	Involved in the study	
☒	☐	ChIP-seq
☒	☐	Flow cytometry
☒	☐	MRI-based neuroimaging

Eukaryotic cell lines

Policy information about cell lines

Cell line source(s)	Human osteosarcoma 143B cells and mouse embryonic fibroblast 3T3 cells were purchased from the American Type Culture Collection (ATCC).
Authentication	Authentication was not performed for this study, because the cell lines were obtained as products from ATCC.
Mycoplasma contamination	All cell lines tested negative for mycoplasma contamination.
Commonly misidentified lines (See ICLAC register)	No commonly misidentified cell lines were used.

Animals and other organisms

Policy information about studies involving animals; ARRIVE guidelines recommended for reporting animal research

Laboratory animals	Adult female Balb/c mice (5-7 weeks, 15–20 g) were obtained from Shanghai Experimental Animal Center of Shanghai Institute of Material Medica, Chinese Academy of Sciences (Shanghai, China). Mice were housed under specific-pathogen-free conditions. The feeding environment is 25°C, 35-45% humidity, 12 h light-dark alternation.

Wild animals	The study did not involve wild animals.
Field-collected samples	The study did not involved samples collected from the field.
Ethics oversight	All operations related to animal experiments follow the relevant requirements of the Institutional Animal Care and Use Committee (IACUC) of Shanghai Institute of Material Medica, Chinese Academy of Sciences.

Note that full information on the approval of the study protocol must also be provided in the manuscript.
